# TDP-43 enhances translation of specific mRNAs linked to neurodegenerative disease

**DOI:** 10.1093/nar/gky972

**Published:** 2018-10-24

**Authors:** Nagammal Neelagandan, Giorgio Gonnella, Stefan Dang, Philipp C Janiesch, Katharine K Miller, Katrin Küchler, Rita F Marques, Daniela Indenbirken, Malik Alawi, Adam Grundhoff, Stefan Kurtz, Kent E Duncan

**Affiliations:** 1 Neuronal Translational Control Research Group, Center for Molecular Neurobiology (ZMNH), University Medical Center Hamburg-Eppendorf (UKE), Hamburg 20251, Germany; 2 Universität Hamburg, MIN-Fakultät, ZBH—Center for Bioinformatics, Hamburg 20146, Germany; 3 Heinrich Pette Institute, Leibniz Institute for Experimental Virology, Hamburg 20251, Germany; 4 Bioinformatics Core, University Medical Center Hamburg-Eppendorf (UKE), Hamburg 20251, Germany

## Abstract

The RNA-binding protein TDP-43 is heavily implicated in neurodegenerative disease. Numerous patient mutations in *TARDBP*, the gene encoding TDP-43, combined with data from animal and cell-based models, imply that altered RNA regulation by TDP-43 causes Amyotrophic Lateral Sclerosis and Frontotemporal Dementia. However, underlying mechanisms remain unresolved. Increased cytoplasmic TDP-43 levels in diseased neurons suggest a possible role in this cellular compartment. Here, we examined the impact on translation of overexpressing human TDP-43 and the TDP-43^A315T^ patient mutant protein in motor neuron-like cells and primary cultures of cortical neurons. In motor-neuron like cells, TDP-43 associates with ribosomes without significantly affecting global translation. However, ribosome profiling and additional assays revealed enhanced translation and direct binding of *Camta1, Mig12*, and *Dennd4a* mRNAs. Overexpressing either wild-type TDP-43 or TDP-43^A315T^ stimulated translation of *Camta1* and *Mig12* mRNAs via their 5′UTRs and increased CAMTA1 and MIG12 protein levels. In contrast, translational enhancement of *Dennd4a* mRNA required a specific 3′UTR region and was specifically observed with the TDP-43^A315T^ patient mutant allele. Our data reveal that TDP-43 can function as an mRNA-specific translational enhancer. Moreover, since CAMTA1 and DENND4A are linked to neurodegeneration, they suggest that this function could contribute to disease.

## INTRODUCTION

TDP-43 is an RNA-binding protein and a major component of ubiquitinated aggregates in motor neurons that are pathological hallmarks of two related neurodegenerative diseases: Amyotrophic Lateral Sclerosis (ALS) and Frontotemporal Dementia (FTD) (1–3). In support of a causal link between altered TDP-43 function and disease, numerous patient mutations have been identified in the *TARDBP* gene, which codes for TDP-43 (4,5). Nevertheless, most patients do not have TDP-43 mutations, suggesting that altered function of wild-type (WT) TDP-43 may be important in these patients. In healthy cells, TDP-43 is primarily localized in the nucleus, whereas in disease it is significantly increased in the cytoplasm, sometimes concomitant with depletion from the nucleus. Altered TDP-43 localization has also been observed in other neurodegenerative diseases, including Alzheimer’s (6), as well as in traumatic brain injury (7).

Disease models based on altered TDP-43 expression in animals and cultured cells have revealed common features of TDP-43 pathophysiology (8). For example, TDP-43′s RNA-binding activity is essential for toxicity (9) and disease-like symptoms do not depend on formation of aggregates per se (10). While other cells are clearly involved in ALS pathology (11), expression of mutant TDP-43 in motor neurons alone can lead to symptoms (12). Moreover, simply overexpressing WT hTDP-43 at a high enough level can lead to disease symptoms (13) and mutant alleles may lead to higher TDP-43 protein levels (14). Collectively, these studies support a model in which altered regulation of one or more cellular RNAs bound by TDP-43 causes disease (15).

Experiments carried out to study the function of TDP-43 have revealed its direct physical RNA targets in specific cell types, including from diseased tissue (16,17). Collectively, these studies reveal a large number of mRNAs that are directly bound by TDP-43 in the nucleus, with relatively fewer in the cytoplasm, consistent with TDP-43 being mainly a nuclear protein. Pinpointing exactly how TDP-43 contributes to disease remains challenging, since TDP-43 binds to so many RNAs and functions in many aspects of mRNA metabolism, including transcription, splicing and stability (16,17). A key unresolved issue is whether disease results from loss of nuclear function, gain of cytoplasmic function, or some combination of the two (18). Several studies show that pre-mRNA splicing is altered in disease, supporting the notion that loss of nuclear TDP-43 and associated effects on splicing would be a major disease driver (16,17,19). However, a later study with new mouse models showed that ALS disease symptoms can occur without any reduction in TDP-43 nuclear levels (10). Interestingly, this study also revealed that mild overexpression of hTDP-43 protein could lead to both loss- and gain-of-function effects on splicing of specific pre-mRNAs and identified mutant-specific events in mice expressing the patient mutant hTDP-43^Q331K^ protein at a similar level to hTDP-43. Nevertheless, despite significant progress, how exactly altered RNA regulation by TDP-43 causes disease remains unclear. The observation that overexpression of either WT or patient variants of TDP-43 in motor neurons can cause disease-like symptoms is consistent with a gain-of-function mechanism. Moreover, the dramatic increase in cytoplasmic TDP-43 levels in affected patient neurons highlights a likely cytoplasmic contribution.

Potential cytoplasmic roles for TDP-43 in disease would include effects on mRNA localization, stability, or translation. In support of a role in localization, axonal mRNA transport rates can be reduced by expression of mutated TDP-43 (20), suggesting that altered mRNA transport could contribute to disease. TDP-43 depletion has been shown to affect levels of many mRNAs in both cultured cells (21) and mouse brain (17). It is not clear for most of these mRNAs whether changes in levels reflect altered transcription or direct effects of TDP-43 on mRNA stability. However, pre-mRNAs with long introns bound by TDP-43 seem to be particularly sensitive to loss of TDP-43, leading to reduced cytoplasmic levels of the corresponding mRNAs (17). The extent to which overexpression of mutant or WT TDP-43 affects mRNA levels is not clear. Although only a few studies have provided evidence for a functional role for TDP-43 in translation, dysregulation of mRNA translation in neurons has been suggested to be important in ALS (22) and is implicated in other forms of neurodegeneration (recently reviewed in (23)). Thus, in principle, cytoplasmic TDP-43 could contribute to neurodegenerative disease by affecting mRNA translation.

Previous studies with cultured cells implicate TDP-43 in translational control. Knocking down TDP-43 in neuronal and non-neuronal cell lines led to a global increase in translation, through an indirect mechanism involving alternative splicing of the exon junction complex component, SKAR (24). In addition, inducing cellular stress with sodium arsenite was reported to lead to TDP-43 association with stalled ribosomes, suggesting that TDP-43 might regulate translation during stress (25). Indirect evidence that cytoplasmic TDP-43 might directly regulate translation of specific mRNAs comes from CLIP-Seq experiments demonstrating that TDP-43 binds to many mRNAs in their 3′UTR, a major region for translational control by RNA-binding proteins (16,17). In *Drosophila*, TDP-43 negatively regulates translation of *futsch* (26) *and hsc 70-4* (27). Two studies in cultured mammalian neurons also provide additional evidence for translational repression by TDP-43 of *Rac1, Map1b* and *GluR1* (*GluA1*) mRNAs (28,29). These directed studies with individual mRNAs indicate that TDP-43 represses translation of specific mRNAs under some circumstances. They also motivate a broader investigation of TDP-43′s impact on translation and how it might contribute to neurodegenerative disease.

Here, we apply genome-wide ribosome profiling to two cell culture models of the cell types affected in ALS and FTD patients: spinal motor neurons and cortical neurons. In combination with several downstream assays, this enabled us to identify three new translational target mRNAs of the human TDP-43^A315T^ mutant: *Camta1, Mig12 and Dennd4a*. Interestingly, CAMTA1 and DENND4A both have strong links to neurodegenerative disease. We show that TDP-43 directly binds to these mRNAs via crosslink-IP, supporting direct regulation. Moreover, overexpressing either WT hTDP-43 or the TDP-43^A315T^ patient mutant protein could stimulate translation of *Camta1* and *Mig12* 5′UTR reporter mRNAs and led to increased levels of endogenous proteins. In contrast, translational enhancement of *Dennd4a* mRNA was only observed with the patient mutant allele via the 3′UTR. Collectively, our data demonstrate a novel function for TDP-43 as an mRNA-specific translational enhancer and further suggest that this function could be an important component of the disease-causing mechanism.

## MATERIALS AND METHODS

### Animal welfare and approvals

All animal care and experimental procedures were performed according to UKE Animal Research Facility institutional guidelines and conformed to the requirements of the German Animal Welfare Act. Relevant approvals: ORG_765 and G14/003_Zucht Neuro.

### Cell line and primary neuronal culture

MN1 cells (30) were grown in Dulbecco’s modified Eagle’s medium high-glucose GlutaMAX culture medium (Gibco), supplemented with 10% fetal bovine serum, 1% penicillin/streptomycin antibiotics and 2.4% HEPES. MN1 cells were transiently transfected with plasmids using Effectene Transfection Reagent (Qiagen) following the manufacturer’s protocol.

Primary neuronal cultures were prepared from cerebral cortices of E16 mice transgenically expressing *Prp-hTARDBP^WT^* (31) or *Prp-hTARDBP^A315T^* (32). Neurons were grown on dishes or glass coverslips coated with poly-L-Lysine in Primary Neuro Basal Medium (Lonza) supplemented with NSF-1, 1% Penicillin/Streptomycin antibiotics and L- Glutamine. 0.5 μM Cytosine β-D-arabinofuranoside (Ara-C) was added to the culture on days *in vitro* 4 (DIV4) to get rid of the cycling cells. Neurons were cultured for 14 days at 37°C in a 5% CO_2_ environment prior to harvesting for ribosome profiling.

### Plasmids and cloning

pcDNA3-FLAG-Mig12 was kindly provided by Prof. Jay Horton, UT Southwestern Medical Center, Dallas, USA. pcDNA3- mycGFP-Mid1 was a kind gift from Dr Germana Meroni, University of Trieste, Trieste, Italy. pEGFP-C1, -C2 or pcDNA3.1 were used as control plasmids, where indicated. The pEGFP-C2 plasmid containing the sequence for ribosomal protein L10a (pEGFP-L10a) was kindly provided by Prof Nathaniel Heintz (Rockefeller University).

The human TDP-43 plasmids used in this study were generated in a pCMV Sport6 vector backbone with an N-terminal FLAG tag and C-terminal V5 tag. pKM29 contains the WT hTDP-43 coding sequence (CDS) in this context and pKM36 has the A315T mutant. The full-length ORF of human TDP-43 was amplified from human TDP-43 (hTDP-43) clone ID30389805 (Open Biosystems) without a stop codon (to allow the addition of 3′ tags to the protein product) and cloned using SalI and NotI into pCMV Sport 6.1. A FLAG-tag-encoding sequence for the 5′ end and a V5-tag-encoding sequence for the 3′ end were made by oligo annealing and cloned using KpnI/SalI (FLAG) and XbaI/HindIII (V5) into the human TDP-43 containing plasmid. The A315T mutation was introduced into the human TDP-43-containing plasmid using the QuikChange Site-Directed Mutagenesis Kit (Agilent Technologies, Cat No: 200519).

pCMV Sport6-Fluc and pCMV Sport6-Rluc for reporter cloning and dual luciferase assays were generated by subcloning the CDS for Firefly or Renilla luciferase from pMT-Fluc or pMT-Rluc (33) into pCMV Sport 6 using KpnI and XhoI sites.

All UTR Renilla luciferase reporter plasmid constructs were generated by cloning into pCMV Sport6- Rluc. UTR sequences were obtained from the ENSEMBL Mouse GRCm38.p6 database. The short 5′UTR of Camta1 was cloned using an oligo annealing technique; 5′UTRs of *Mig12* and *Dennd4a* were polymerase chain reaction (PCR) amplified from MN1 cDNA and all 5′UTR constructs were cloned with 5′ AvrII and 3′ KpnI restriction sites. The 3′UTR of *Camta1* was PCR amplified from mouse genomic DNA and cloned with 5′ NotI and 3′ MluI restriction sites. 3′UTRs of *Mig12* and *Dennd4a* were PCR amplified from mouse genomic DNA and cloned with 5′ XhoI and 3′ MluI restriction sites. All plasmid sequences were confirmed by Sanger sequencing using at least two primers per plasmid. Cloning primers are listed in Supplementary Table S9.

### Ribosome profiling

The original protocol (34) was followed with few modifications. MN1 cells were treated with 100 μg/ml cycloheximide (CHX) for 3 min before lysis. No CHX treatment was done for primary neurons prior to lysis. Cells were lysed with lysis buffer containing 20 mM Tris–HCl, pH 7.4, 150 mM NaCl, 5 mM MgCl_2_, 1 mM dithiothreitol (DTT) and 100 μg/ml CHX (added freshly) supplemented with 1% (vol/vol) Triton X-100 and 25 U/ml Turbo DNase(Ambion). To lysate containing 1000 μg total protein, 0.075 μl RNaseI (Ambion) was added. Samples were incubated at 25°C in a shaking thermomixer for 45 min. Reactions were stopped by adding 1.5 μl SUPERaseIN (Invitrogen). During RNaseI incubation, fresh sucrose solutions with 50 and 17.5% sucrose were prepared in buffer containing 20 mM Tris–HCl, pH 7.4, 150 mM NaCl, 5 mM MgCl_2_, 1 mM DTT 100 μg/ml CHX and 20 U/ml SUPERaseIN. The sucrose gradients were generated using the Gradient Master 108 programmable gradient pourer (Biocomp). Samples were then loaded onto sucrose gradients and centrifuged for 2.5 h at 35 000 rpm in a SW40Ti rotor in a Beckman L7 ultracentrifuge (Beckman Coulter). After centrifugation, gradients were fractionated and measured for RNA content using a Piston Gradient Fractionator (Biocomp) attached to a UV monitor (BioRad). Fractions containing monosome peaks were selected for footprint library preparation.

RNA extraction was done using sodium dodecyl sulphate (SDS), acid phenol and PCI followed by isopropanol precipitation. Total RNA was poly(A) selected and fragmented. Both total RNA and footprints were run on a 15% TBE/Urea/polyacrylamide gels (Life Technologies) and size selected by loading 28mer and 34mer marker mix. The fragment of defined size was cut out and library generation was performed as described previously (34). The libraries were multiplexed sequenced on an Illumina HiSeq2500 SR 50 base run.

### Analysis of the ribosome profiling data

For the analysis of the ribosome profiling data, we implemented the open source pipeline Ribopip (http://github.com/stepf/RiboPip). The pipeline is implemented in Ruby and follows the protocol of (34), with minor differences described here.

For all analyses, we used the mouse genome sequence and annotation GRCm38.p4 as a reference. Adapter clipping was performed using cutadapt 1.8.1 (35) with the parameters: –trimmed-only -e 0. No filtering regarding the read length was applied. Non-coding RNAs were removed by filtering out the reads which bowtie2 (36) aligned to the annotated non-coding RNAs, using a seed length and a minimum alignment length of 14.

The alignment of the remaining reads to the genome was performed using TopHat2 (37) with Bowtie2 (36) as read mapper. For all alignments, we set a minimum seed length parameter to 14. To compensate for the different lengths of the remaining reads, we decided to allow a number of mismatches in the alignment to the genome, relative to the read length. However, this option is not provided by the alignment tools we used. Therefore, we split, prior to the alignment, the reads into buckets of reads of equal length. Each bucket of reads was aligned separately, allowing a maximum number of ceil(e*l) mismatches, where e is the desired error rate (we set e to 0.1) and l is the read length. If e*l is not an integer, we round to the smallest integer larger than e*l. After the computation, the alignments of each bucket were joined.

The number of hits for each annotated feature was determined using FeatureCounts 1.4.4 (38). DeSeq (39) was used for the analysis of the differential expression between samples.

Details of read counts and mapping for each sample are in Supplementary Table S1. Mapping to UTRs versus CDS appear in Supplementary Table S2.

### TRAP immunoprecipitations from MN1 cells

MN1 cells in 10-cm dishes were transfected with either pEGFP-C2 or pEGFP-C2-L10a alone or co-transfected with FLAG-hTDP-43-V5 using Effectene reagent (Qiagen) according to the manufacturer’s protocol. Twenty-four hours after transfection, cells were collected and spun down at 1000 *g* for 10 min at 4°C. Cells were then lysed in buffer containing 10 mM Tris HCl, pH 8.0, 150 mM NaCl, 10 mM MgCl_2_, 0.25% NP-40, 0.1% Triton-X 100, 1× Complete mini ethylenediaminetetraacetic acid (EDTA)-free protease cocktail (Roche), 0.1 U/ml RNasin, 20 mM DTT and 200 μg/ml CHX. Lysates were spun at 20 000 × *g* for 10 min at 4°C and supernatants were mixed with beads pre-bound to anti-GFP monoclonal antibodies (clones 19C8 and 19F7, Sloan Kettering MAb Facility), as follows. Antibodies were thawed on ice, then spun at maximum speed in a microfuge for 10 min at 4°C, transferred to a new tube and 0.02% sodium azide was added. Streptavidin MyOne T1 Dynabeads (Invitrogen/Life Technologies) were resuspended by hand mixing, and 300 μl beads were transferred to a microfuge tube. The tube was placed in the provided magnet for 1 min, and supernatant was removed. Beads were resuspended in 1 ml phosphate-buffered saline (PBS). Beads were then incubated in 120 μl of Biotinylated Protein L (Thermo scientific) for 35 min at RT using gentle end over end rotation. The coated beads were then washed 5× with PBS containing 3% (weight/volume) of IgG and protease-free bovine serum albumin (Jackson Immunoresearch). A total of 100 μg of anti-GFP antibody per reaction was added (50 μg of HtZ-GFP 19C8 and 50 μg of HtZ-GFP 19F7) in 1 ml ‘0.15 M KCl buffer’ (20 mM HEPES, 5 mM MgCl2, 150 mM KCl, 1% NP-40, 0.5 mM DTT and 100 μg/ml CHX). Beads were incubated at RT for 1 h, rotating slowly. Tubes were placed on the magnet for 1 min and supernatant was removed. Beads were washed 3× in 1 ml 0.15 M KCl buffer, resuspended in 200 μl 0.15 M KCl and stored on ice. Cleared lysates and beads were incubated overnight at 4°C with end over end rotation and beads were collected on the magnet on ice, and supernatant was removed. Beads were washed 4× in 1 ml 0.35 M KCl IP wash buffer (20 mM HEPES, 5 mM MgCl2, 350 mM KCl, 1% NP-40, 0.5 mM DTT and 100 μg/ml CHX). One time Laemmli protein loading buffer was added directly to the beads, and beads were boiled at 95°C for 5 min to elute, supernatants were collected and used for sodium dodecyl sulphate-polyacrylamide gel electrophoresis (SDS-PAGE).

### Polysome profiling

**Figure 1.**
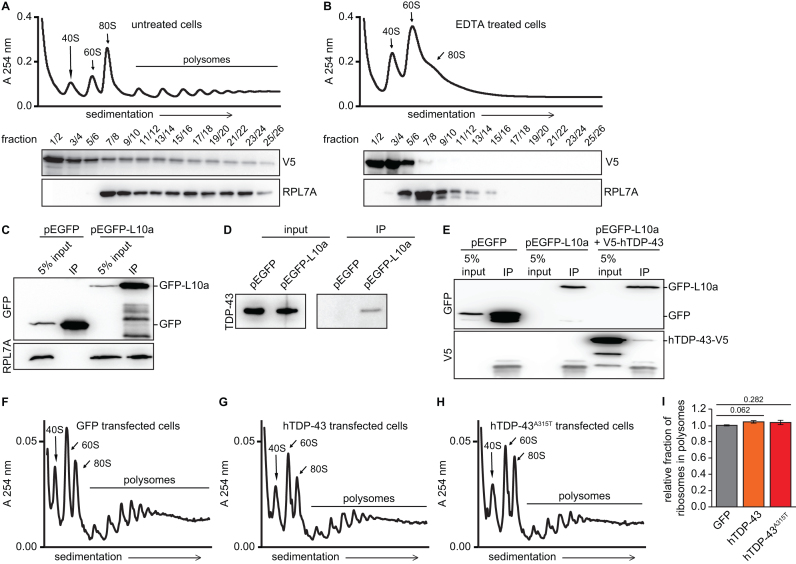
TDP-43 associates with polyribosomes in cells that are actively translating. (**A** and **B**) Representative polysome profiles are shown for MN1 cells transiently transfected with hTDP-43-V5 under either standard conditions (A) or after EDTA treatment to disrupt polysomes (B). Immunoblots for hTDP-43-V5 and ribosomal protein L7a in the indicated pooled gradient fractions are aligned to the corresponding gradients. Note the clear hTDP-43-V5 signal in deep polysome fractions under standard conditions that shifts to lighter fractions upon EDTA treatment. (**C**–**E**) MN1 cells were transfected with either pEGFP or pEGFP-L10a and used for immunoprecipitation with anit-GFP antibodies under conditions for TRAP. Immunoblotting of input and IPs reveals that ribosomal protein L7a (C), endogenous TDP-43 (D) and co-transfected TDP-43-V5 (E) all co-immunoprecipitate specifically with GFP-L10a, but not with GFP alone. (**F**-**H**) Representative polysome profiles from MN1 cells transiently transfected with GFP (F) hTDP-43-V5 (G) or hTDP-43^A315T^-V5 (**H**) are shown. Note the similarity of the profiles. (**I**) Quantification of the relative fraction of ribosomes in polysomes from independent experiments reveals no statistically significant difference (*n* = 3, s.e.m. error bars, *P*-value indicated, unpaired two-tailed *t*-test).

For Figure 1A and 1B, one third of a 70–90% confluent 10 cm dish was re-plated to a new 10 cm dish to achieve between 40 and 80% confluency upon transfection. MN1 cells were transfected in 10 cm dishes with FLAG-hTDP-43-V5 using Effectene reagent (Qiagen) following the manufacturer’s protocol. Forty-eight hours later, cells were treated with 50 μg/ml CHX for 30 min before lysis. Two 10 cm dishes were pooled for each gradient sample and cells were lysed by incubation for 10 min on ice in buffer containing 20 mM Tris HCl, pH 7.4, 100 mM NaCl,10 mM MgCl_2_, 0.4% NP-40, 50 μg/ml CHX, 1× Complete mini EDTA-free protease cocktail (Roche) and 100 U/ml RNaseIn. After lysis, nuclei were pelleted by a quick spin and the post-nuclear supernatant was spun at 10 000 × *g* for 10 min at 4°C. Cleared lysate supernatant was transferred to a new tube and Bradford assays were performed and used to normalize gradient loading. For Figure 1B, EDTA was added directly to the cell lysates (final concentration 15 mM) prior to centrifugation. Fresh sucrose solutions with 50 and 17.5% sucrose were prepared in buffer containing 10 mM Tris–HCl, 75 mM KCl and 1.5 mM MgCl_2_. A total of 17.5–50% sucrose gradients were generated using the Gradient Master 108 programmable gradient pourer (Biocomp) and chilled at 4°C for at least 30 min prior to use. Ultracentrifugation and fractionation were performed as described above for ribosome profiling. For protein isolation for immunoblotting, gradient fractions were pooled according to the scheme presented in Figure 1A and 1B, concentrated by TCA precipitation and resuspended in 1× Laemmli Buffer for SDS-PAGE.

For data in Figure 3 and Supplementary Figure S2, MN1 cells were transfected with GFP, FLAG-hTDP-43-V5 or FLAG-hTDP-43^A315T^-V5 for 24 h and treated with 50 μg/ml CHX for 3 min before lysis. Transfected MN1 cells or cultured neurons were lysed in buffer containing 20 mM Tris HCl, pH 7.4, 100 mM NaCl, 5 mM MgCl2, 0.4% NP-40, 50 ug/ml CHX, 1× Complete mini EDTA-free protease cocktail (Roche) and 2.5 μl RNaseIn/ml lysis buffer. Cells were lysed on ice for 10 min and cell debris was then spun down at maximum speed for 10 min at 4°C. Protein concentrations were determined by Bradford assays and used to normalize gradient loading. Lysates were loaded onto 17.5–50% sucrose gradients in buffer containing 20 mM Tris HCl, pH 7.4, 100 mM NaCl, 5mM MgCl_2_, 0.4% NP-40, 50 μg/ml CHX, Protease Inhibitor, 20 U/ml SUPERaseIN and fractionated as described for ribosome profiling. For RNA isolation, fractions were pooled according to the scheme presented in Figure 3 and processed as described below. A fraction of the original lysate loaded onto the gradient was retained and processed in parallel as a reference for total cytoplasmic RNA.

### Analysis of the fraction of ribosomes in polysomes

The area under the curves representing the monosome and polysome peaks in gradient profiles were quantified using ImageJ (40). The fraction of ribosomes in polysomes was calculated by dividing polysome area by the sum of polysome and monosome areas.

### RNA extraction from polysome gradients and qRT-PCR

Gradient fractions were pooled according to the scheme presented and RNA was isolated from the pooled fractions using Trizol in a ratio of 3:1 followed by the addition of chloroform and subsequent purification by PureLink kit (Ambion). A total of 450 ng of RNA was used to make cDNA in each fraction. cDNA libraries were generated using SuperScript^®^ II Reverse Transcriptase (Life Technologies) according to the manufacturer’s protocol for random hexamer priming. FastStart Universal SYBR Green Master ROX (Roche) was used for qRT-PCR with three technical replicates per sample and reactions were run on ABI 7900HT instruments. The standard curve method was used for analysis with ABI instrument software followed by export to Microsoft Excel. To obtain relative distribution plots, 18S rRNA levels were measured and their relative distribution across the fraction pools were calculated. The same was done for all other RNAs studied and their distribution was additionally normalized to the one obtained for 18S RNA and expressed as a percentage of cumulative signal.

### Crosslink immunoprecipitation (CLIP)

MN1 cells were seeded in 10 cm dishes 2 days prior to performing the assay and were ∼70–80% confluent when used. Culture medium was removed and ice-cold 1× PBS was added to the cells followed by UV irradiation (200 mJ/cm^2^) using a Stratalinker. Cross-linked cells were lysed in 1 ml of Lysis Buffer (50 mM Tris–HCl, pH 7.4, 100 mM NaCl, 1% NP-40, 0.1% SDS, 0.5% Na-deoxycholate, 1× Complete Protease Inhibitor Cocktail (Roche)). A fraction of the lysate corresponding to 5% of the input material (50 μl) was retained to use as a reference for calculating fraction of input material in the IP pellet. The remaining lysate was added to Protein G Dynabeads pre-bound with either 2.5 μg rabbit polyclonal TDP43 antibody (abcam ab41881) or rabbit IgG (Jackson Immuno) as a control and rotated at 4°C overnight. Beads were subsequently washed twice for 2 min in High Salt Buffer (50 mM Tris–HCl, pH 7.4, 1 M NaCl, 1 mM EDTA, pH 8.0, 1% NP-40, 0.1% SDS, 0.5% Na-deoxycholate) followed by washing twice for 2 min in wash buffer (20 mM Tris–HCl, pH 7.4, 10 mM NaCl, 0.2% Tween-20) and a final washing step for 2 min in NT2 buffer (50 mM Tris–HCl, pH 7.4, 150 mM NaCl, 1 mM MgCl_2_, 0.05% NP-40). For protein analysis, 1× or 6× Laemmli buffer was added directly to the beads or input fraction, respectively, followed by incubation at 95°C for 5 min. RNA was eluted by incubation with 30 μg Proteinase K (Carl Roth) in NT2 Buffer for 30 min at 55°C. RNA extraction was carried out from the eluate and input sample using Trizol reagent as described earlier. All RNA obtained from each sample (Input or IP) was used to generate cDNA libraries using random hexamers (Thermo Scientific) and the RevertAid RT reverse transcription kit (Thermo Scientific), following the manufacturer’s protocol.

To calculate target mRNA enrichments, we first calculated the delta *C*_t_ for TDP-43 IP versus Input and converted this to a linear ‘Fold Change’ value. These were then corrected for the reduced amount of input analyzed (i.e. divided by 20), and then multiplied by 100 to obtain ‘% of Input mRNA in IP’. Supplementary Table S7 shows the *C*_t_ values for each sample and all subsequent analyses for the three replicates used to generate the data in Figure 4C and D.

### qRT-PCR primers

**Table:** 

mRNA	Forward primer (5′-3′)	Reverse primer (5′-3′)
*Camta1*	ATTCTGCGGAACTAGCACCT	ATTTCGGCAGACATTCAAGC
*Dennd4a*	GTCAGGGCTCTGAAAACTGC	GTCCACAGAGCTGCATGAGA
*Mig12*	AGCACTGAGAACCAGGGGAT	GGCTGCTCTTATCTTTGGCT
*Gapdh*	TTGATGGCAACAATCTCCAC	CGTCCCGTAGACAAAATGGT
*Pth1r*	CACTAAGCTTCGGGAGACCA	GGCCATGAAGACGGTGTAGT
*Tardbp*	CGTGTCTCAGTGTATGAGAGGAGTC	CTGCAGAGGAAGCATCTGTCTCATCC
*18s rRNA*	CTTAGAGGGACAAGTGGCG	ACGCTGAGCCAGTCAGTGTA
*Rluc*	TGGTAACGCGGCCTCTTCT	GCCTGATTTGCCCATACCAA
*Fluc*	GTTTCCAAAAAGGGGTTG	CATCGACTGAAATCCCTGGT

### Immunostaining

Transfected cells or neurons were grown on glass cover slips coated with poly L-Lysine. Twenty-four hours after transfection for MN1 and on DIV2 for primary neurons, cells were fixed with 4% PFA for 2 min followed by ice cold methanol for 3 min and three washes with 1× PBS. Blocking was done using 5% goat serum in 1× PBS. The following primary antibodies were used: rabbit polyclonal CAMTA1 (Sigma-HPA036343) (1:100), rabbit polyclonal MID1IP1 (Sigma- HPA038816) (1:100), mouse monoclonal V5 (Invitrogen- 46–1157) (1:100), mouse monoclonal Tau1 (Millipore- MAB3420) (1:100) and mouse monoclonal Acetylated-Tubulin (Sigma- T7451) (1:750). Coverslips were incubated with primary antibodies in blocking solution at 4°C overnight. Coverslips were then washed three times with 1× PBS and incubated with secondary antibodies in blocking solution at room temperature for 2 h in dark. Coverslips were then washed three times with 1× PBS, submerged in MilliQ water and mounted on glass microscope slides with Fluoromount-G (Southern Biotech). Cells were imaged using an Olympus Fluoview 1000 microscope with 60× objective, using similar acquisition settings for laser power, offset and detector gain across conditions.

### Microscopy image analysis

Image analysis was done using Fiji (41). Transfected cells were marked with V5 (for hTDP-43 / hTDP-43^A315T^) and/or GFP (for GFP and MID1-GFP) as markers. The region of interest was marked with DAPI for nuclear staining. For cytoplasmic staining, the Nuclear DAPI staining region was masked in the original image and residual mean intensity for the whole cell region was calculated. For primary neurons, the mean intensity gray value of a line drawn along the neurites marked using acetylated tubulin or Tau1 was measured using Fiji. Linear adjustments of brightness and contrast were performed on images using Photoshop CS.

### Reporter transfection, dual luciferase assays and determination of mRNA levels

Transient transfections in MN1 cells were performed in 24-well plates using Effectene Transfection Reagent (Qiagen) according to the manufacturer’s protocol. The amount of plasmids used were GFP/Flag-hTDP-43-V5/Flag-hTDP-43^A315T^-V5- 180 ng/170 ng; Rluc ctrl/Camta1 5′UTR-Rluc/ Camta1 3′UTR- Rluc- 17 ng; Mig12 5′UTR-Rluc/ Mig12 3′UTR-Rluc/ Dennd4a 5′UTR-Rluc/Dennd4a 3′UTR- Rluc – 9 ng; Fluc- 11 ng/13 ng. Cells were lysed by adding 150 μl 1× Passive Lysis Buffer (Promega, Cat No: E1910) per well of a 24-well plate and incubating in a shaking platform for 15 min. Lysate was spun down in a microfuge at maximum speed for 1 min and 10 μl of supernatant for each sample was loaded in duplicate to 96-well luminometer plates. The plate was measured in a Victor3 (TM) 1420 Multilabel counter luminometer (Perkin Elmer), set to dispense 50 μl of each Dual Luciferase Assay reagent (Promega, Cat No: E1910) per well.

For RNA extraction from transfected reporter samples, lysates were treated with Turbo DNase (Ambion), to get rid of plasmid-derived signal prior to preparing RNA with Trizol reagent, as described above. A total of 250 ng of RNA was used to make cDNA for these experiments. The protocol was as described above for crosslink immunoprecipitation (CLIP), except at the cDNA synthesis step, each sample was divided in two and incubated either in the presence or absence of Reverse Transcriptase (‘no RT’), to verify that signal was due to mRNA and not contaminating DNA. qPCR was performed as described above and analyzed using the Δ*C*_t_ method. All samples used for analysis showed clear enrichment compared to the corresponding no RT control.

### Western blotting

Immunoblotting to nitrocellulose or polyvinylidene difluoride (PVDF) was performed either with wet transfer under standard conditions or using an iBlot rapid transfer device (Life Technologies) according to the manufacturer’s guidelines. Blots were blocked in 5% milk/TBS-T solution and probed with antibodies diluted as indicated. Signals were either visualized using HRP-conjugated secondary antibodies and Super Signal Dura or Femto reagent (Thermo Fisher Scientific) and imaged on a Fujifilm LAS-4000 luminescent image analyzer or by using fluorescent secondary antibodies and imaged on a Li-Cor Odyssey CLx (Li-Cor). Total protein staining was performed using Revert Total Protein Stain Kit (Li-Cor) according to the manufacturer’s instructions and used for normalization. Antibodies used in this study were rabbit anti-human polyclonal MID1IP1 (Sigma- HPA038816) (1:250), mouse monoclonal V5 (Invitrogen- 46-1157) (1:1000), rabbit polyclonal ribosomal protein L7a (E109)(CST-2415) (1:1000), anti-human monoclonal TDP-43 (Novus biologicals- H000023435-M01) (1:500), rabbit polyclonal TDP-43 (G400) (CST-3448) (1:1000), mouse monoclonal GAPDH (Sigma- G8795) (1:1000- 1:2000), mouse monoclonal FLAG (clone M2, Sigma- F1804) (1:1000), mouse monoclonal α-Tubulin (Sigma-Aldrich T5168), rabbit polyclonal Cofilin (Abcam-ab42824) (1:2000), rabbit monoclonal S6 ribosomal protein (5G10) (CST- 2217), goat anti-rabbit HRP (1:10 000, Thermo Fisher Scientific), goat anti- mouse HRP (1:10 000, Thermo Fisher Scientific), goat anti-rabbit IRDye 680LT (1:15 000, Li-Cor), goat anti- mouse IRDye 680LT (1:15 000, Li-Cor), goat anti-rabbit IRDye 800CW (1:15000, Li-Cor), goat anti- mouse IRDye 800CW (1:15 000, Li-Cor). Western blot quantification was done using ImageJ or Image studio™ Lite (LI-COR Biosciences).

### MIG12 and CAMTA1 knockdown

ON-TARGET plus Mouse *Mid1ip1* siRNA-SMART pool (L-063562-01-0005) and ON-TARGETplus Mouse Camta1 siRNA-SMART pool (L-051054-00-0005) was purchased from GE Healthcare/Dharmacon. siRNA transfections were performed using X-tremeGENE siRNA Transfection reagent (Roche) according to the manufacturer’s protocol. MN1 cells were grown on poly-L-Lysine coated coverslips for immunostaining as described above for immunostaining. For RNA extraction, cells grown on 24-well plates were washed with 1× PBS and Trizol was directly added onto them. This was followed by the addition of chloroform and subsequent purification by PureLink kit according to the manufacturer’s protocol (Ambion). cDNA libraries were generated using RevertAid RT reverse transcription kit (Thermo Scientific) as described above.

### Statistical analysis and plots

All statistical tests were performed in GraphPad Prism (version 5.02) or Microsoft Excel. Two-tailed unpaired *t*-tests were performed, unless otherwise indicated. Plots were generated either using GraphPad Prism (version 5.02), OriginPro 2017G or Microsoft Excel. Venn diagrams were generated online using Venny: http://bioinfogp.cnb.csic.es/tools/venny/.

## RESULTS

### TDP-43 can associate with polyribosomes in cells that are actively translating

TDP-43 was found to associate with ribosomes during acute cellular stress induced by sodium arsenite, which leads to massively reduced translation of most mRNAs (25). Whether TDP-43 can also associate with ribosomes in unstressed cells that are actively translating is unknown. To determine whether TDP-43 interacts with ribosome–mRNA complexes in actively translating cells, we first performed polysome profiling with the motor neuron-like cell line, MN1 (30). We transiently transfected these cells with human TDP-43 (hTDP-43) bearing a V5 epitope-tag on the C-terminus, prepared polysome gradients and used immunoblotting to analyze how transfected TDP-43 protein distributed across the gradients. Under standard conditions, we detected a significant fraction of TDP-43-V5 that co-migrated with the large ribosomal subunit protein, L7a, in deep polysome fractions (Figure 1A). Treatment of lysates with the Mg^2+^ chelator EDTA prior to centrifugation led to the expected total disruption of polysomes into 40S and 60S subunits and completely shifted ribosomal protein L7a to lighter gradient fractions corresponding to the large ribosomal subunit (Figure 1B). EDTA treatment also shifted TDP-43-V5 signal from polysomal fractions to lighter mRNP fractions, consistent with TDP-43 association with polyribosome–mRNA complexes.

To verify TDP-43 association with ribosome–mRNA complexes in an independent assay, we examined whether it co-purified with ribosomes in translating ribosome affinity purification (TRAP), which preserves polyribosome–mRNA complexes (42). We performed TRAP from MN1 cells transfected with plasmids encoding a GFP-L10a fusion protein or GFP as a negative control. Both GFP-L10a and GFP were efficiently immunoprecipitated, but co-immunoprecipitation of the large ribosomal subunit protein L7A was only observed with GFP-L10a, supporting efficient and specific immunoprecipitation of ribosomes (Figure 1C). Similarly, both endogenous TDP-43 and transfected TDP-43-V5 specifically interacted with GFP-L10a, but not with GFP alone (Figure 1D and E). Taken together, our results from polysome and TRAP assays imply that a fraction of TDP-43 protein associates with ribosome–mRNA complexes in the absence of cellular stress when translation is actively occurring.

### Overexpression of human TDP-43 or TDP-43^A315T^ protein does not lead to a significant effect on general translation

TDP-43’s association with ribosomes in actively translating cells suggested a potential role in translational regulation. In principle, this could be general or mRNA-specific. Therefore, we first tested whether overexpression of WT human TDP-43 (hTDP-43) or a patient mutant protein (hTDP-43^A315T^) affected general translation in motor neuron-like cells. Polysome profiles from MN1 cells transfected with either hTDP-43 or hTDP-43^A315T^ appeared similar to GFP-transfected control profiles (Figure 1F–H), suggesting no significant impact of TDP-43 overexpression on general translation. Indeed, calculating the fraction of ribosomes in polysomes from multiple experimental replicates revealed no statistically significant differences among the different transfections (Figure 1I). Thus, TDP-43 overexpression does not significantly affect general translation in motor neuron-like cells.

### Ribosome profiling reveals potential translational targets of TDP-43 in MN1 cells and primary neurons

**Figure 2.**
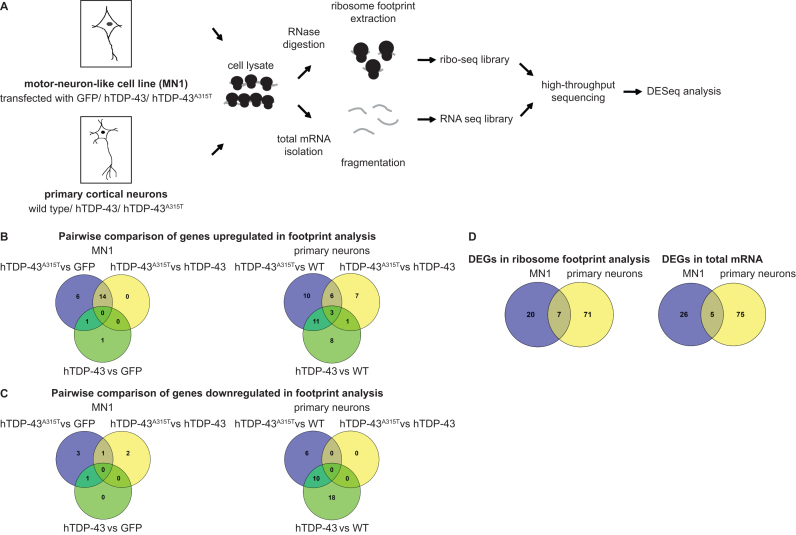
Identification of potential translational targets of hTDP-43 and hTDP-43^A315T^ by ribosome profiling from motor neuron-like cells and primary cortical neurons. (**A**) Overview of experimental design. MN1 cells were transfected with GFP control or human TDP-43 variants (hTDP-43-V5 or hTDP-43^A315T^-V5) (*n* = 2). Primary cortical neurons were prepared from E16 WT mice (*n* = 2) or mice expressing human TDP-43 variants (hTDP-43 and Flag-hTDP-43^A315T^, *n* = 3). Neurons were grown in culture for 2 weeks (DIV14). Ribosome footprints and total mRNA were extracted from each of these cell types, followed by library generation and high-throughput sequencing. (**B** and **C**) Overview of differentially regulated genes. Venn diagrams show pairwise comparisons of genes that were upregulated (B) or down regulated (C) in MN1 and primary cortical neurons. (**D**) Venn diagrams showing differentially expressed genes (DEGs) identified in MN1 cells, primary cortical neurons, or both. DEGs from ribosome footprint analysis (translation) and total mRNA sequencing (transcription/mRNA stability) are shown separately.

To identify specific mRNAs regulated by TDP-43 at the translational level, we performed ribosome profiling with two different cell populations: motor neuron-like MN1 cells and primary cortical neuronal cultures (Figure 2A). For MN1 cells, we performed two replicates, using cells transiently transfected with plasmids encoding GFP, hTDP-43 or the mutant hTDP-43^A315T^ protein found in a subset of ALS patients. Importantly, expression levels of TDP-43 variants were similar and total TDP-43 levels in these assays suggest quite moderate overexpression (Supplementary Figure S1A–C). Primary cortical neurons were obtained from hemizygous transgenic animals expressing either hTDP-43 or hTDP-43^A315T^ mutant and non-transgenic littermate control animals. Mutant protein levels in primary cultures were somewhat higher than the WT hTDP-43, but total overexpression levels were not especially high (Supplementary Figure S1D and E). Because only the mutant animals develop disease as hemizygotes (31,32), hTDP-43 neurons serve as an important control. Changes specifically observed in cortical neurons expressing hTDP-43^A315T^, but not hTDP-43, correlate with disease. We followed the standard protocol for generating ribosome footprint profiling libraries and also generated libraries in parallel from total mRNA to control for transcriptional versus translational regulation (34). Details of sequencing library preparation, sequencing and data analysis are described in the ‘Materials and Methods’ section. Supplementary Table S1 provides an overview of the mapping statistics and read counts obtained for each individual sample. Exon-mapped read numbers and mapping rates were similar to other published ribosome profiling experiments (43,44). Importantly, we also observed the expected preference in read mapping to CDS versus 3′UTRs in ribosome footprint samples, but not corresponding total mRNA samples (Supplementary Table S2).

To identify differentially expressed mRNAs, we performed three different comparisons for each cell type: GFP/WT versus hTDP-43, hTDP-43 versus hTDP-43^A315T^ and GFP/WT versus hTDP-43^A315T^ (Supplementary Tables S3–S6). To identify altered translation, we refined our hits to genes that show altered regulation only in the ribosome footprints, but not in total mRNA (Figure 2B and C). Strikingly, we identified a set of mRNAs whose translation was altered by hTDP-43^A315T^ in both MN1 cells and primary cortical neurons (Figure 2D and Table 1). These genes are especially interesting, as they show changes in both cell populations that are affected in the disease. Thus, we have identified potential new translational targets of hTDP-43 and hTDP-43^A315T^ proteins in MN1 cells and primary cortical neurons.

**Table 1.** Genes found to be differentially regulated by hTDP-43^A315T^ expression in both MN1 cells and primary cortical neurons

DEGs found in both primary cortical neurons and MN1 cells in ribosome footprint analysis and the comparison(s) where these genes were identified.			
Ensembl ID	Gene symbol	MN1	Primary cortical neuron
ENSMUSG00000052117	*D630039A03Rik*	hTDP-43 A315T versus hTDP-43 WT, hTDP-43 A315T versus WT, hTDP- 43WT versus WT,	GFP versus hTDP-43 A315T
ENSMUSG00000032492	*Pth1r*	hTDP-43 A315T versus hTDP-43 WT, hTDP-43 A315T versus WT	hTDP-43 WT versus hTDP-43 A315T, GFP versus hTDP-43 A315T
ENSMUSG00000008035	*Mid1ip1/Mig12*	hTDP-43 A315T versus hTDP-43 WT, hTDP-43 A315T versus WT	GFP versus hTDP-43 A315T
ENSMUSG00000095738	*Gm25313*	hTDP-43 A315T versus hTDP-43 WT	GFP versus hTDP-43 A315T
ENSMUSG00000029797	*Sspo*	hTDP-43 A315T versus hTDP-43 WT	GFP versus hTDP-43 A315T
ENSMUSG00000046854	*Pip5kl1*	hTDP- 43WT versus WT	GFP versus hTDP-43 A315T
ENSMUSG00000027552	*E2f5*	hTDP-43 A315T versus WT	GFP versus hTDP-43 A315T, hTDP-43 WT versus hTDP-43 A315T


DEGs found in both primary cortical neurons and MN1 cells in total mRNA analysis and the comparison(s) where these genes were identified.			
Ensembl ID	Gene symbol	MN1	Primary cortical neuron
ENSMUSG00000030428	*Ttyh1*	hTDP-43 A315T versus WT	hTDP-43 WT versus GFP
ENSMUSG00000028195	*Cyr61*	hTDP-43 A315T versus WT	hTDP-43 WT versus hTDP-43 A315T
ENSMUSG00000031216	*Stard8*	hTDP-43 A315T versus WT	hTDP-43 WT versus hTDP-43 A315T
ENSMUSG00000018916	*Csf2*	hTDP-43 A315T versus WT	hTDP-43 WT versus hTDP-43 A315T, hTDP-43 WT versus GFP
ENSMUSG00000034883	*Lrr1*	hTDP-43 A315T versus WT	hTDP-43 WT versus hTDP-43 A315T

To prioritize translational targets from ribosome profiling for further experiments, we first focused on mRNAs that showed altered translational regulation only in response to the patient mutant hTDP-43^A315T^ protein. Even though overexpression of hTDP-43 WT at a sufficiently high level can cause disease-like phenotypes (45), we know the level of expression of hTDP-43-WT in the transgenic animals that we used for primary neurons is not sufficient to cause disease (31) (Supplementary Figure S1D). Moreover, total TDP-43 expression levels in our transfected MN1 cells are also not especially high (Supplementary Figure S1A), suggesting that this filter might identify targets more likely to be disease-relevant in both systems. After applying this filter, we next selected genes for follow-up analysis that met one of two criteria: either (i) they showed strong, direct links to neurodegenerative disease based on previous studies, or (ii) they were identified in both cell types. Based on these criteria, we identified four genes for further downstream analysis: *Camta1, Dennd4a, Pth1r* and *Mig12/Mid1ip1* (Table 2).

**Table 2.** Genes selected from ribosome profiling for further validation

Ensembl ID	Gene symbol	Gene name
ENSMUSG00000014592	*Camta1*	Calmodulin-binding transcription activator 1
ENSMUSG00000053641	*Dennd4a*	DENN domain containing 4A
ENSMUSG00000008035	*Mid1ip1/Mig12*	Mid1 interacting protein 1
ENSMUSG00000032492	*Pth1r*	Parathyroid hormone 1 receptor

### Polysome profiling confirms that hTDP-43^A315T^ protein increases ribosome density on *Camta1, Mig12* and *Dennd4a* mRNAs in MN1 cells

**Figure 3.**
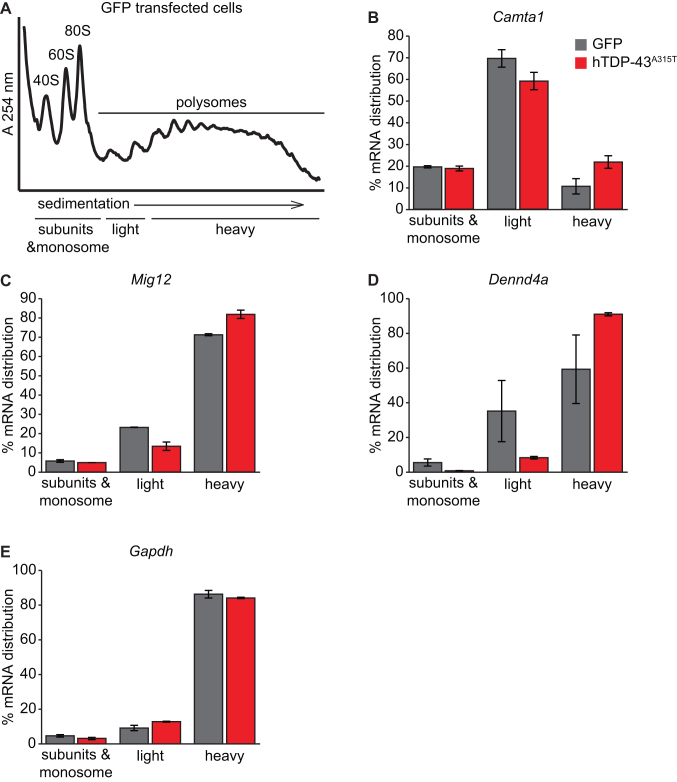
Polysome profiling confirms that TDP-43^A315T^ regulates translation of specific mRNAs. (**A**) Representative polysome profile of control MN1 cells transfected with GFP. The pooling scheme of gradient fractions used for mRNA distribution analysis is indicated. For the purpose of analysis, the distribution was separated into (i) subunits and monosomes, (ii) light polysomes (2–3 ribosomes/mRNA) and (iii) heavy polysomes (≥4 ribosomes/mRNA). (**B**–**E**) Plots showing mRNA distribution for candidate mRNAs identified by ribosome footprint profiling: *Camta1* (B), *Mig12* (C), *Dennd4a* (D) and *Gapdh* (E). (*n* = 2; values normalized to 18s rRNA; error bars show deviation from average in the replicates).

Like all high-throughput methods, ribosome profiling can lead to false positives. Therefore, we used an independent, orthogonal assay to validate the effects of hTDP-43^A315T^ on translation of specific mRNAs that we identified by ribosome profiling. Specifically, we performed sucrose density-gradient polysome profiling of specific mRNAs with transiently transfected MN1 cells. We pooled polysome gradient fractions as indicated (Figure 3A), purified RNA, and examined the relative levels of specific mRNAs in pooled gradient fractions by qRT-PCR in two independent validation experiments. When hTDP-43^A315T^ protein was expressed, *Camta1, Mig12* and *Dennd4a* mRNAs all shifted to heavier gradient fractions (Figure 3B–D), implying increased ribosome density on these mRNAs, exactly as seen in our genome-wide ribosome profiling experiments (Supplementary Tables S3 and 4). In contrast, the distribution of *Gapdh* mRNA in polysomes was not affected by hTDP43^A315T^ in these experiments, highlighting a specific effect on translation of the other mRNAs (Figure 3E). *Pth1r* mRNA also did not show a shift in this analysis, suggesting that it might have been a false positive (Supplementary Figure 2D). We did not observe a significant change in total mRNA levels for *Camta1, Mig12 or Dennd4a* mRNAs (Supplementary Figure S2E–H). Taken together, these results confirm that exogenous expression of the hTDP-43^A315T^ protein increases ribosome density on *Camta1, Mig12* and *Dennd4a* mRNAs and therefore affects their translation in motor neuron-like cells.

### TDP-43 protein interacts directly with *Camta1, Mig12* and *Dennd4A* mRNAs

**Figure 4.**
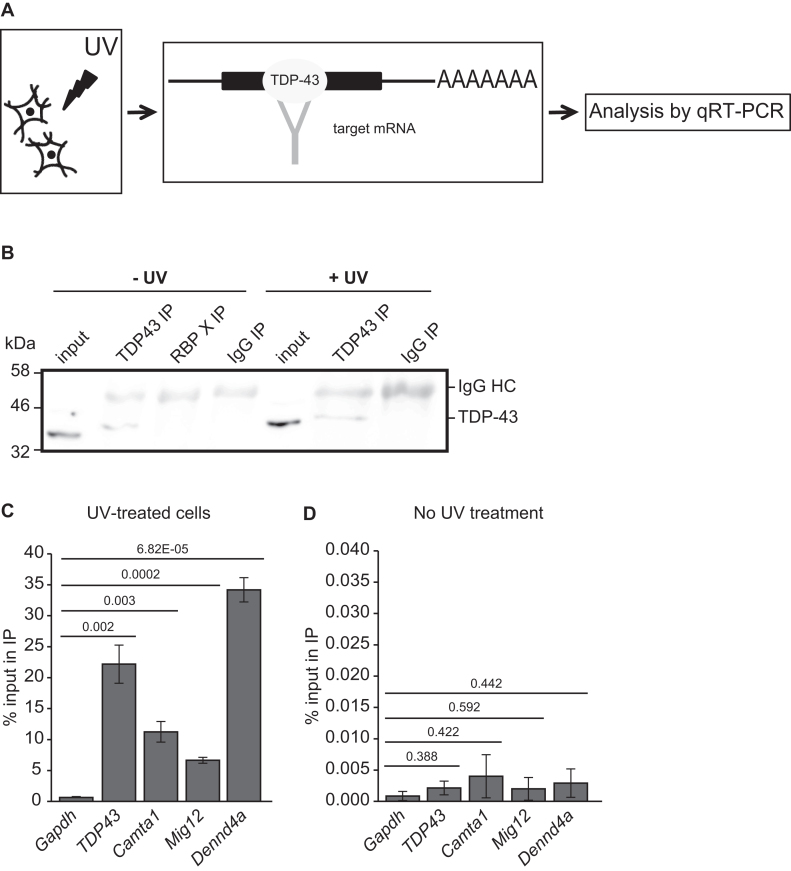
CLIP shows that TDP-43 directly interacts with translational target mRNAs. (**A**) Schematic diagram of the CLIP experiment. (**B**) Immunoblot showing specific IP of TDP-43 from MN1 cells under CLIP conditions. A TDP-43 band is specifically observed in the input and TDP-43 IP lanes from both untreated and UV-treated cells, but not in the IgG or anti-RBP X control lanes. RBP X is an IP of a different RNA-binding protein, which serves as an additional specificity control here. (**C** and **D**) Plots showing percentage of input mRNA in the TDP-43 IP for the mRNAs indicated in three separate CLIP assays from MN1 cells. Performing CLIP from UV-treated cells leads to significant recovery and enrichment relative to *Gapdh* for all mRNAs examined (C). In the absence of UV treatment, much less mRNA is recovered and no significant enrichment relative to *Gapdh* mRNA is observed. Note that the *Y*-axis scale in D is 1000× smaller than in C, indicating strong crosslinking dependence of mRNA co-immunoprecipitation (*n* = 3, s.e.m. error bars, *P*-values indicated in the plot, unpaired two-tailed *t*-test).

In principle, hTDP-43^A315T^ could affect translation of specific mRNAs through direct or indirect mechanisms. To distinguish between these possibilities, we used UV-CLIP, to determine whether TDP-43 can interact directly with these mRNAs in MN1 cells (Figure 4A). We first confirmed specific immunoprecipitation of endogenous TDP-43 protein with anti-TDP-43 antibody relative to control IPs and that IP efficiency was not affected by UV treatment (Figure 4B). Next, we used qRT-PCR to examine levels of specific mRNAs in the different IP samples, comparing *Camta1*,*Mig12* and*Dennd4a* mRNAs to *Tdp-43* and *Gapdh* mRNAs. *Tdp-43* mRNA serves as a positive control, since TDP-43 is known to bind to its own mRNA for autoregulation (46). Conversely, biologically significant binding of *Gapdh* mRNA by TDP-43 has not been described. We first confirmed that mRNA signals in TDP-43 IPs from UV-treated cells were greater than IgG control background in each of our three replicate experiments (Supplementary Table S7). Next, we quantified the percent of input mRNA that co-immunoprecipitated with TDP-43 protein. In UV-treated cells, we observed significant enrichment over *Gapdh* mRNA for *Tdp-43, Camta1, Mig12 and Dennd4a* mRNAs (Figure 4C). In the absence of UV treatment, mRNA recovery was reduced by more than 100- fold and significant enrichment relative to *Gapdh* mRNA was lost, demonstrating crosslink dependence of the detected interactions. (Figure 4D and Supplementary Table S7). We conclude that TDP-43 protein interacts directly with *Camta1, Mig12*, and*Dennd4a* mRNAs in motor neuron-like MN1 cells.

### hTDP-43 and hTDP-43^A315T^ enhance translation of *Camta1* and *Mig12* mRNAs via their 5′UTRs

We next sought to determine which regions of the translationally targeted mRNAs might be important for regulation. First, we manually searched for potential TDP-43 binding sites in annotated *Camta1, Mig12* and *Dennd4a* mRNAs. Long (UG) repeats strongly correlate with TDP-43 binding, but shorter motifs are neither necessary nor sufficient (16,17). We found no repeats >5 nt in these mRNAs (Supplementary Table S8), suggesting that TDP-43 either recognizes these mRNAs through short motifs or other types of binding sites.

**Figure 5.**
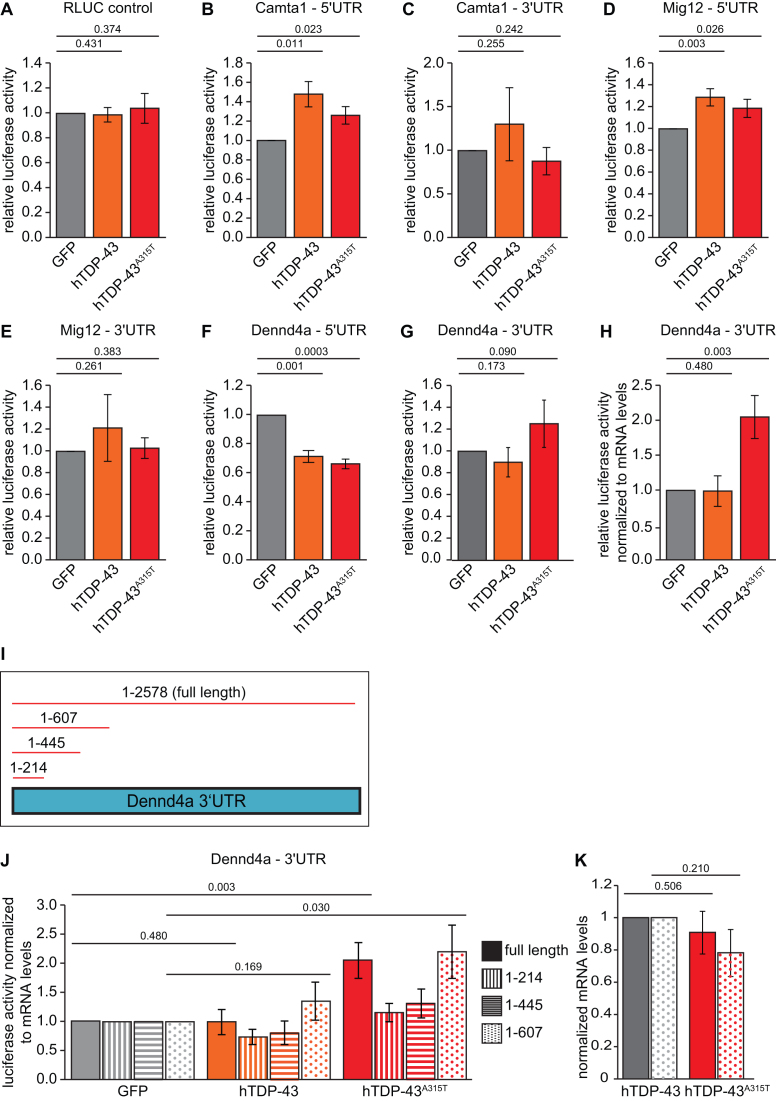
Luciferase reporter assays reveal mRNA regions important for translational regulation by WT and mutant TDP-43. Relative luciferase activity measured by dual luciferase assay in MN1 cells transfected with GFP, hTDP-43 or hTDP-43^A315T^ and co-transfected with Fluc and either Rluc vector ctrl containing no UTR of interest (**A**), *Camta1* 5′UTR (**B**), *Camta1* 3′UTR (**C**), *Mig12* 5′UTR (**D**), *Mig12* 3′UTR (**E**), *Dennd4a* 5′UTR (**F**), *Dennd4a* 3′UTR (**G**). (**H**) Relative luciferase activity normalized to mRNA levels in cells co-transfected with *Dennd4a* 3′UTR. (**I**) Schematic of *Dennd4a* 3′UTR-derived constructs used for this study. (**J**) Relative luciferase activity normalized to mRNA levels in cells co-transfected with GFP, hTDP-43 or hTDP-43^A315T^, Fluc, and the indicated Dennd4a 3′UTR constructs shown in I (*n* = 3–7; s.e.m. error bars, *P*-value indicated, unpaired one-tailed *t*-test). (**K**) Comparison of hTDP-43 mRNA expression levels in the MN1 cells transiently co-transfected with hTDP-43 or hTDP-43^A315T^. Lack of a significant difference implies that specific regulation of the *Dennd4a* reporters by hTDP-43^A315T^ is not due to differential expression versus hTDP-43 (*n* = 3–7; s.e.m. error bars, *P*-values indicated, unpaired two-tailed *t*-test).

Next, we generated plasmids encoding Renilla luciferase (RLuc) reporters with 5′ or 3′UTRs from *Mig12*,*Camta1* and*Dennd4a* mRNAs. We co-transfected MN1 cells with these reporters and an excess of a plasmid encoding hTDP-43^A315T^ or an equivalent amount of a control plasmid encoding GFP. In parallel, we also evaluated whether the effects were indeed specific for the mutant TDP-43 protein by co-transfecting a similar amount of plasmid encoding hTDP-43, which we know gives similar protein expression levels to the mutant under our assay conditions (Supplementary Figures S1A–C and 2A–C). As shown in Figure 5, in dual luciferase assays we observed enhanced RLuc levels only from specific combinations of reporters and proteins. Importantly, under the tested conditions neither hTDP-43 nor hTDP-43^A315T^ affected RLuc production from the control plasmid used for cloning (Figure 5A). Co-transfection of either hTDP-43 or hTDP-43^A315T^ led to a significant increase in Rluc activity for both *Camta1* and *Mig12* 5′UTR reporters (Figure 5B and D). In contrast, no significant increase was observed for *Camta1* or *Mig-12* 3′UTR reporters with either protein (Figure 5C and E). These data highlight the 5′UTRs of *Camta1* and *Mig12* mRNAs as important regions for their translational enhancement by TDP-43. They also reveal that regulation in these cases is not specific to the patient mutant allele: hTDP-43 and hTDP-43A315T were both able to stimulate RLuc output from these reporters to similar extents.

### hTDP-43^A315T^ enhances translation of *Dennd4a* mRNA through a mechanism that requires a specific 3′UTR region

Unlike *Camta1* and *Mig12*, the 5′UTR of *Dennd4A* mRNA appeared to confer translational repression by both hTDP-43 and hTDP-43^A315T^ under our assay conditions (Figure 5F). This was surprising, given that hTDP-43^A315T^ expression increased ribosome density on *Dennd4a* mRNA in both polysome assays (Figure 3D) and the original ribosome profiling screen (Supplementary Table S3). Standard luciferase measurements with the *Dennd4a* 3′UTR reporter showed only a trend toward increased RLuc activity in the presence of hTDP-43^A315T^ (Figure 5G). However, normalizing luciferase activity to reporter mRNA levels revealed a significant increase in RLuc protein/mRNA that was strikingly specific for the hTDP-43^A315T^ mutant protein (Figure 5H). These data suggest that a disease-causing allele of TDP-43 has a function not shared with WT TDP-43: selective enhancement of translation of *Dennd4a* mRNA via its 3′UTR.

To determine which regions of the *Dennd4a* 3′UTR might be important for selective translational enhancement by hTDP-43^A315T^, we tested a series of truncation mutants in the same assay setup (Figure 5I and J). As shown in Figure 5J, hTDP-43^A315T^ was able to enhance translation of a reporter containing nucleotides 1–607 of the *Dennd4a* mRNA 3′UTR to a level comparable to the full-length 3′UTR reporter, whereas truncating further abolished regulation. As expected, WT hTDP-43 did not stimulate translation of any of these reporters. Importantly, this was not due to differential expression of hTDP-43, since its mRNA levels were very similar to those for hTDP-43^A315T^ in these assays (Figure 5K). These data suggest that a region between nucleotides 445 and 607 of the *Dennd4a* 3′UTR is particularly important for selective translational enhancement of this mRNA by a patient mutant allele of TDP-43.

### hTDP-43 overexpression leads to increased CAMTA1 and MIG12 protein levels in MN1 cells

Having established that TDP-43 binds directly to *Camta1, Mig12* and *Dennd4a* mRNAs and can enhance translation via their UTRs, we next sought to determine the potential functional impact of altered translation of these mRNAs on levels of the encoded proteins. After testing a number of antibodies in both western blot and immunofluorescence applications, we identified antibodies to CAMTA1 and MIG12 that gave strong signal in immunofluorescence assays. Moreover, this signal was specific, since it was significantly reduced upon siRNA knockdown of the corresponding mRNA (Supplementary Figure S3). For this reason, we focused our analysis on these two proteins, using immunofluorescence signal intensity as a readout for protein levels. In addition, we also reasoned that immunostaining might be more sensitive and could provide information about potential spatial effects of altered protein synthesis. We transfected MN1 cells with V5-tagged hTDP-43 variants and examined the impact on endogenous CAMTA1 and MIG12 immunofluorescence signal intensity in confocal microscopy images. Transfection efficiencies were around 70% in these assays, allowing us to compare protein levels in transfected (V5^+^) cells to neighboring untransfected (V5^−^) cells from the same coverslip. Since these untransfected (V5^−^) cells have been processed and imaged in parallel, they serve as an ideal internal control.

**Figure 6.**
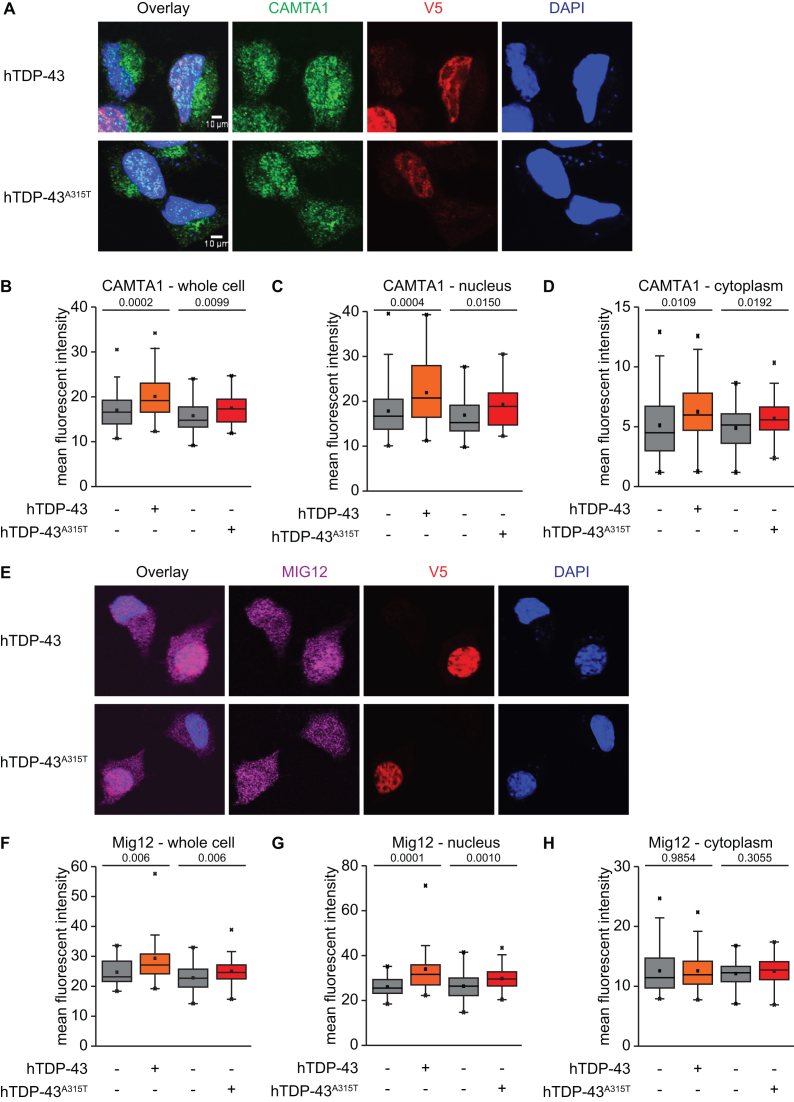
hTDP-43 or hTDP-43^A315T^ overexpression leads to increased CAMTA1 and MIG12 protein levels in MN1 cells. (**A**) Immunofluorescence of MN1 cells transiently transfected with hTDP-43-V5 or hTDP-43^A315T^-V5 and stained for CAMTA1 (green), V5 (red) and DAPI (blue); Scale bar represents 10 μm. Intensity of CAMTA1 in whole cell (**B**), nucleus (**C**) and cytoplasm (**D**); (*n* = 40–60 cells each; *P*-value indicated in each plot; unpaired two-tailed *t*-test). (**E**) Immunofluorescence of MN1 cells transiently transfected with hTDP-43-V5 or hTDP-43^A315T^-V5 and stained for MIG12 (magenta), V5 (red) and DAPI (blue); Scale bar represents 10 μm. Intensity of MIG12 in whole cell (**F**), nucleus (**G**) and cytoplasm (**H**); (*n* = 30–70 cells each; *P*-value indicated in each plot; unpaired two-tailed *t*-test).

Consistent with the reporter assay results, we observed increased levels of CAMTA1 protein in cells transfected with either hTDP-43 WT or hTDP-43^A315T^ protein (Figure 6A and B) and this was manifested in both the nucleus and cytoplasm (Figure 6C and D). MIG12 protein levels were also increased in cells transfected with either hTDP-43-V5 or hTDP-43^A315T^–V5 protein relative to neighboring untransfected (V5^−^) control cells (Figure 6E and F). However, a significant increase in MIG12 protein levels appeared only in the nucleus, even though specific MIG12 protein signal was present in both the nucleus and cytoplasm (Figure 6G and H; Supplementary Figure S3). As an additional control for these studies, we transfected a plasmid encoding EGFP instead of TDP-43 and compared GFP^+^ cells to their GFP^−^ neighbors. CAMTA1 and MIG12 protein levels were not changed between GFP^+^ and GFP^−^ cells (Supplementary Figure S4), highlighting a specific role for TDP-43 in this assay. These data indicate that increased levels of TDP-43 can enhance protein levels encoded by two of its directly bound translational targets. Moreover, as for translational enhancement of *Camta1* and *Mig12* 5′UTR reporters in luciferase assays (Figure 5), these effects on endogenous protein levels are observed with either WT or mutant TDP-43.

### hTDP-43^A315T^ expression leads to increased MIG12 protein levels in processes of primary cortical neurons

**Figure 7.**
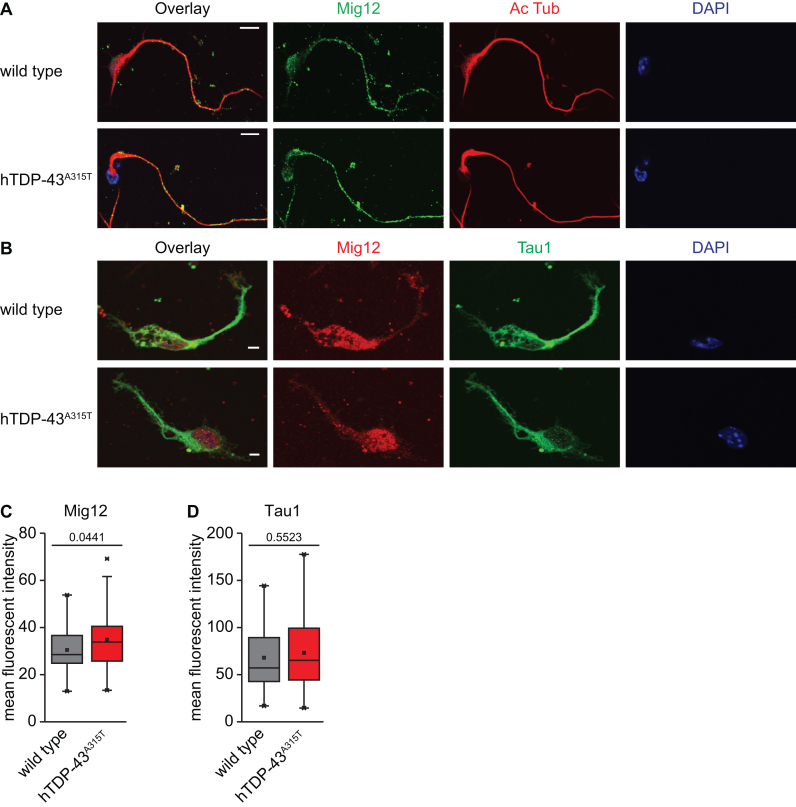
hTDP-43^A315T^ expression leads to increased MIG12 protein levels in processes of primary cortical neurons. (**A** and **B**) Representative immunofluorescence images of DIV2 primary cortical neurons from mice expressing the hTDP-43^A315T^ protein or non-transgenic WT littermates. Scale bar represents 10 μm. (A) MIG12 (green), Acetylated tubulin (red) and DAPI (blue). (B) MIG12 (red), TAU (using Tau1 antibody, red) and DAPI (blue); (**C**) Quantification of mean fluorescent intensity of MIG12 in neurites. (*n* = 37–40 cells from three independent cultures; unpaired two-tailed *t*-test, *P*-values indicated in plot). (**D**) Quantification of mean fluorescent intensity of Tau1 immunoreactivity in neurites (*n* = 30–33 cells from three independent cultures; unpaired two-tailed *t*-test, *P*-values indicated in plot).

In our ribosome profiling screen, MIG12 was identified as an hTDP-43^A315T^ target in both MN1 cells and primary neuronal cultures (Table 1). Thus, we next investigated the effect of mutant hTDP-43 expression on MIG12 protein levels in primary cortical neurons derived from the same transgenic mouse ALS model expressing hTDP-43^A315T^ (32). As in MN1 cells, MIG12 was present in both the nucleus and cytosol of cultured cortical neurons. Moreover, we also detected MIG12 staining in neuronal processes marked by Tau1 antibody staining (Figure 7A and B). In contrast to MN1 cells, we did not observe any change in nuclear MIG12 intensity in cultured cortical neurons expressing hTDP-43^A315T^ relative to non-transgenic littermate controls (Supplementary Figure S5). However, quantification of MIG12 signal intensity in neuronal processes revealed a significant increase in the MIG12 protein level in neurites of cells expressing hTDP-43^A315T^ protein as compared to non-transgenic WT cells (Figure 7C). In contrast, levels of phosphorylated Tau protein (Tau1 staining) in neuronal processes were not significantly altered (Figure 7B and D), supporting a specific effect on MIG12 protein levels. Thus, our immunostaining data show that MIG12 protein levels are also affected by hTDP-43^A315T^ protein in primary cultures of cortical neurons. Intriguingly, the effect here is also cellular compartment-specific, but manifests in neuronal processes, rather than in the nucleus.

### MID1, a disease-associated MIG12 binding partner, modifies TDP-43’s effects on MIG12 protein

**Figure 8.**
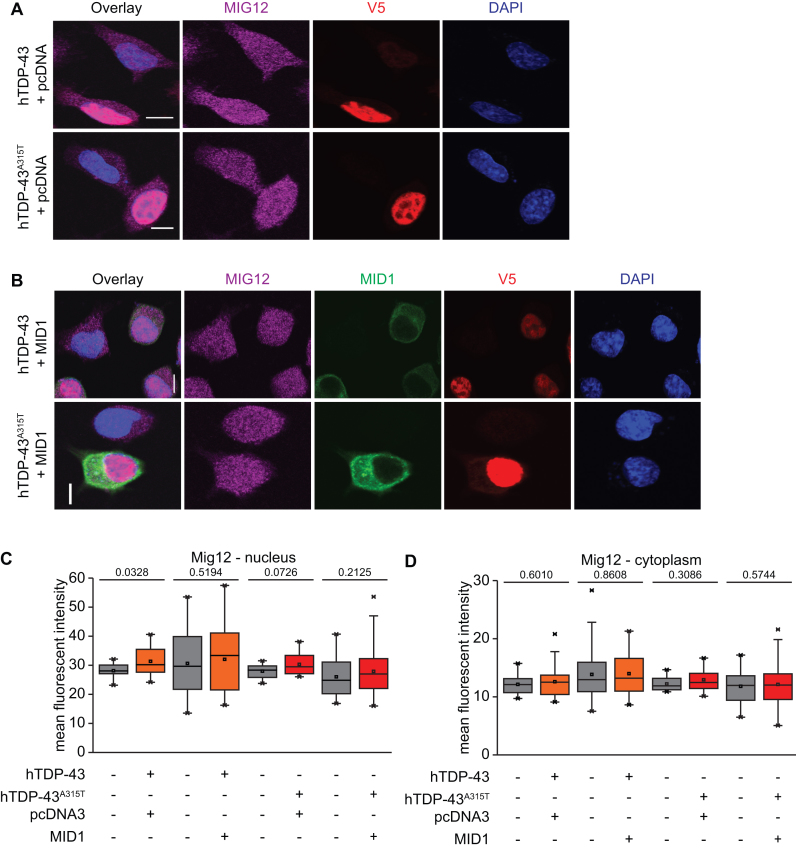
Overexpressing the MIG12 binding partner, MID1, abolishes TDP-43’s effects on MIG12 protein levels. Immunofluorescence of MN1 cells transiently transfected with (**A**) Flag-hTDP-43- V5/ Flag- hTDP-43^A315T^-V5 and pcDNA control plasmid stained for MIG12 (Magenta), V5 (red) and DAPI (blue). Immunofluorescence of MN1 cells transiently transfected with (**B**) Flag-hTDP-43- V5/ Flag- hTDP-43^A315T^-V5 and MycGFP Mid1 plasmid stained for MIG12 (Magenta), MID1 (green from GFP), V5 (red) and DAPI (blue). Scale bar represents 10 μm. Intensity of MIG12 in nucleus (**C**) and cytoplasm (**D**) (*n* = 10–24 cells for hTDP-43/ hTDP-43^A315T^ and pcDNA3 transfection; *n* = 42–72 cells for hTDP-43/hTDP-43^A315T^ and MycGFP Mid1 transfection; *P*-value indicated in each plot; unpaired two-tailed *t*-test).

Unlike CAMTA1 and DENND4a, no direct connection between MIG12 and neurodegenerative disease has previously been described. However, several lines of evidence support a role for MIG12’s protein-protein interaction partner, MID1, in both neurodevelopmental disorders and neurodegenerative disease (47). MID1 is a cytoplasmic protein whose molecular functions include E3 ubiquitin ligase activity and regulation of translation through direct and indirect mechanisms (47–51). This suggests that MID1 binding to MIG12 could affect MIG12 protein levels, and that this could be a potential connection between altered *Mig12* mRNA translation and neurodegenerative disease. To examine this possibility, we asked whether increasing cytoplasmic MID1 levels had any impact on how TDP-43 affects MIG12 protein levels. Specifically, we transfected MN1 cells with either hTDP-43 or hTDP-43^A315T^ and co-transfected equivalent amounts of either pcDNA3 empty vector control or myc-GFP-MID1 (Figure 8A and B, respectively). When pcDNA3 empty vector was co-transfected, we observed the expected increased protein levels driven by hTDP-43 or hTDP-43^A315T^ (Figure 8A and C). In striking contrast, co-transfection of a myc-GFP-MID1 plasmid led to expression of myc-GFP-MID1 in the cytoplasm and completely abolished TDP-43’s effects on nuclear MIG12 levels (Figure 8B and C). Cytoplasmic MIG12 levels were not affected by hTDP-43 or hTDP-43^A315T^ when either pCDNA3.1 or myc-GFP-MID1 was co-transfected (Figure 8D). Thus, overexpression of MID1 can mitigate the effect of overexpressing hTDP-43 proteins on MIG12 protein levels in MN1 cells. These results suggest that cytoplasmic MID1 levels can determine whether altered ribosome density on *Mig12* mRNA leads to altered MIG12 protein levels in the nucleus.

## DISCUSSION

TDP-43 has a central role in the neurodegenerative diseases ALS and FTD, but how exactly it causes these diseases remains unclear. Studies to date have focused more on loss of function and the impact of overexpressed TDP-43 on altered nuclear functions. Less attention has been paid to potential direct effects on cytoplasmic steps of gene expression. Here, we used genome-wide ribosome profiling of motor neuron-like cells and primary cortical neurons to examine the effects of overexpressing WT hTDP-43 and the patient mutant hTDP-43^A315T^. In combination with several downstream assays, this revealed a novel function of TDP-43 as an mRNA-specific translational enhancer for *Camta1, Mig12* and*Dennd4a*. Interestingly, overexpressing either WT or the TDP-43^A315T^ patient mutant protein stimulated translation of *Camta1* and *Mig12* mRNAs. In contrast, translational enhancement of *Dennd4a* mRNA was only observed with the patient mutant allele.

How does TDP-43 enhance translation? For *Camta1* and *Mig12* mRNAs, our data suggest a gain-of-function mechanism operating through their 5′UTRs. Overexpressing either WT or mutant hTDP-43 similarly affected luciferase reporters derived from these mRNAs, as well as cellular levels of CAMTA1 and MIG12 proteins in MN1 cells. Our luciferase reporter data also highlight the 5′UTRs of these mRNAs as a key *cis*-element for their translational enhancement by TDP-43. Taken together with our CLIP data demonstrating that TDP-43 binds directly to both mRNAs, this suggests that TDP-43 functions through the 5′UTRs of these mRNAs to stimulate translation. Manual inspection of these UTRs does not reveal obvious similarities, raising the possibility that the underlying mechanisms might nevertheless be different, despite both involving 5′UTRs. Translational enhancement of *Dennd4a* mRNA appears to involve a fundamentally different mechanism. In this case, overexpression of the hTDP-43^A315T^ mutant, but not WT hTDP-43, selectively enhanced translational output via the *Dennd4a* 3′UTR. Moreover, using CLIP, we found that WT TDP-43 can be efficiently crosslinked to this mRNA. This suggests that differential translational enhancer activity, rather than binding per se underlies the mutant-specific effect. In the future, a systematic comparison of the impact of other patient alleles on *Dennd4a* translation would be interesting. It will also be important to map the exact regions that mediate TDP-43 interaction with these mRNAs and compare in detail the translational enhancer mechanisms operating via their UTRs.

Previous studies suggest how TDP-43’s translational enhancer function might be relevant to neurodegenerative disease. Mice lacking *Camta1* develop ataxia and show Purkinje cell degeneration along with significant cerebellar atrophy at the age of 3 months (52). In humans, *CAMTA1* variants are implicated in neurobehavioral abnormalities (53), episodic memory performance (54) and modulate ALS patient survival (55). Moreover, both CAMTA1 and DENND4A were independently found to be ‘Master Regulators’ (MRs) of neurodegenerative disease transcriptional programs in an in vivo Parkinson’s disease model and a cultured motor neuron-based ALS model (56,57). Remarkably, CAMTA1 and DENND4A were among the three common MRs identified in both studies. This suggests a central role for both CAMTA1 and DENND4A proteins in driving transcriptional programs underlying neurodegenerative diseases. The ALS study also showed that DENND4A is a motor neuron ‘death driver’, since *Dennd4a* mRNA knockdown led to significantly enhanced survival of cultured spinal motor neurons. This suggests that enhanced translation of *Dennd4a* mRNA might promote motor neuron death. It is unclear what upstream events trigger deregulation of the Master Regulators themselves to unleash the transcriptional program that drives disease. Because mRNA levels for the MRs do not change in disease, the trigger presumably is post-transcriptional. Our data raise the possibility that altered translation of mRNAs encoding neurodegeneration Master Regulators could be an important, relatively early component of disease caused by altered TDP-43 activity in the cytoplasm.

According to our results, any physiological condition that leads to gain of TDP-43 function could affect *Camta1* mRNA translation. What though would be the significance for disease of a mutant-specific effect on *Dennd4a* mRNA translation, given that most ALS does not involve mutations in TDP-43? Similar to cancer, where oncogene expression is rarely sufficient on its own to cause disease, neurodegenerative diseases are hypothesized to follow a multiple hit model (58). Although the ‘hits’ are not completely defined, one idea is that specific lesions combine with chronic activation of cellular stress pathways to push cells over a ‘stressor threshold’ (59). Thus, chronic stress (or some other ‘hit’) might ultimately lead to deregulated DENND4A expression in ALS patients lacking a TDP-43 mutation. In principle, this could involve regulatory effects on WT TDP-43 that we do not model in our MN1 cell assays. Alternatively, DENND4A activation in the absence of TDP-43 mutations could occur through a TDP-43-independent mechanism or might not even be necessary in some forms of ALS. Our results motivate *in vivo* experiments to examine these scenarios and dissect the potential contribution of altered translation of *Camta1* and *Dennd4A* mRNAs to disease onset and progression.

In the case of MIG12, a potential connection between enhanced translation by TDP-43 and disease is less obvious. In MN1 cells, overexpression of either hTDP-43 or hTDP-43^A315T^ led to increased nuclear protein, whereas in primary cortical neurons, hTDP-43^A315T^ overexpression led to increased MIG12 protein levels only in neurites. Taken together, these results highlight dynamic regulation of MIG12 protein levels in response to TDP-43 overexpression, which varies with cell type. What might be the physiological consequence of altered MIG12 levels in these different compartments? MIG12’s nuclear function has yet to be identified, but two cytoplasmic functions for MIG12 have been described: regulating fatty acid synthesis via effects on the acetyl-CoA carboxylase complex (60) and binding Mid1 to affect microtubule stabilization (61). Changes in fatty acid metabolism (62) and microtubule dynamics are both implicated in neurodegeneration (63,64). Thus, our work motivates future use of ALS models to test whether altered MIG12 protein levels are indeed affecting these processes and to evaluate their potential contributions to disease phenotypes.

Our results also illustrate general principles of how mRNA-specific translational control affects protein levels. First, the impact of altered mRNA translation on levels of the encoded protein can be cell-compartment specific. While this is intuitive for local translation in neuronal processes, it is less obvious that effects on translation would only affect nuclear protein levels for a protein also present in the cytoplasm. Nevertheless, this is what we see for MIG12 in MN1 cells. Second, which compartment is affected can be cell-type specific: unlike cultured MN1 cells, we saw increased MIG12 in primary cortical neuron processes. Third, whether altered translation affects protein levels at all can be modulated by changes in levels of that protein’s interaction partners. Overexpressing MIG12’s cytoplasmic binding partner, MID1, abolished a TDP-43-driven increase in nuclear MIG12 protein levels in motor neuron-like cells. Since MID1 is an E3 ubiquitin ligase (48,50,51), this could imply that newly synthesized MIG12 is rapidly targeted by MID1 for degradation by the ubiquitin-proteasome system. While MID1 has been implicated in RNA regulation of key molecules in Alzheimer’s and Huntington’s disease (reviewed in (47)), it has not previously been implicated in TDP-43-driven neurodegeneration. Our data suggest it would be interesting to see how MID1 is regulated in ALS/FTD and how this may relate to TDP-43’s effects on *Mig12* mRNA translation.

In sum, we have identified a new function for TDP-43 as an mRNA-specific translational enhancer. TDP-43 gain-of-function can enhance translation of *Camta1* and *Mig12* mRNAs in motor neuron-like cells via mechanisms operating through their 5′UTRs. In contrast, we found that translational enhancement of *Dennd4a* mRNA occurs via the 3′UTR and is selectively triggered by a mutant allele of TDP-43 that causes disease in patients. Our results help to elucidate translational regulation by RNA-binding proteins and raise the possibility that TDP-43’s mRNA-specific translational enhancer function contributes to neurodegenerative disease.

## DATA AVAILABILITY

Ribopip is an open source pipeline for the analysis of the ribosome profiling data implemented in Ruby and available in the GitHub repository (http://github.com/stepf/RiboPip).

BAM Files from this study mapped to the UCSC browser are available.

Ribosome Profiling and RNA-Seq datasets from this study are available through the Gene Expression Omnibus (GEO) under accession number GSE111775.

## Supplementary Material

Supplementary Data
