# Supplementary material for: TDP-43 enhances translation of specific mRNAs linked to neurodegenerative disease
Source: Nucleic Acids Res. 2018 Oct 24;47(1):341–61. doi: 10.1093/nar/gky972 (PMC6326785; doi:10.1093/nar/gky972)
Supplement: Supplementary Data [file gky972_supplemental_files.zip › SUPPLEMENTARY MATERIAL- Neelagandan et al-NAR-02745-R2.pdf]

SUPPLEMENTARY FIGURE 1 - Neelagandan et al

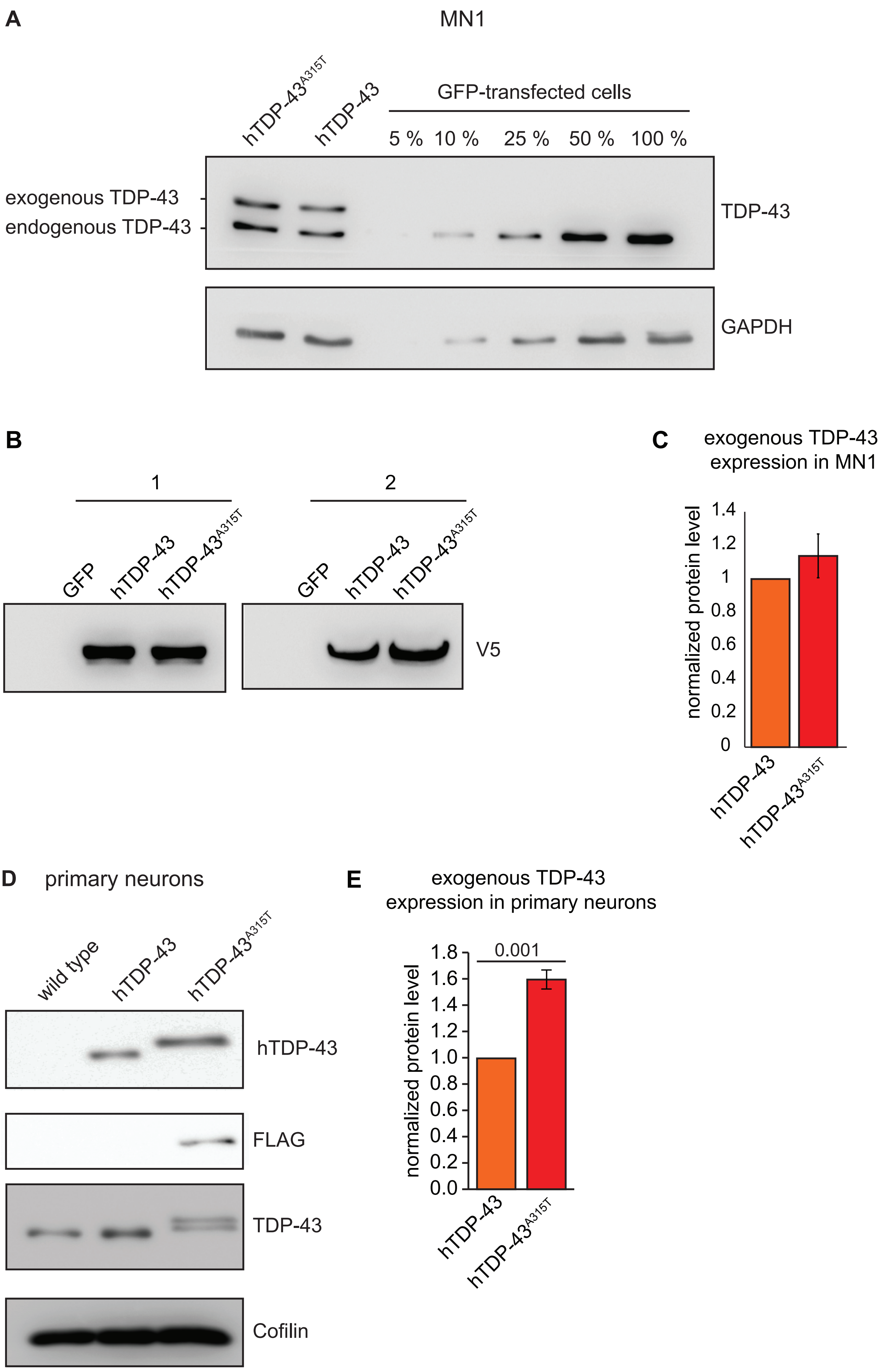

SUPPLEMENTARY FIGURE 2 - Neelagandan et al

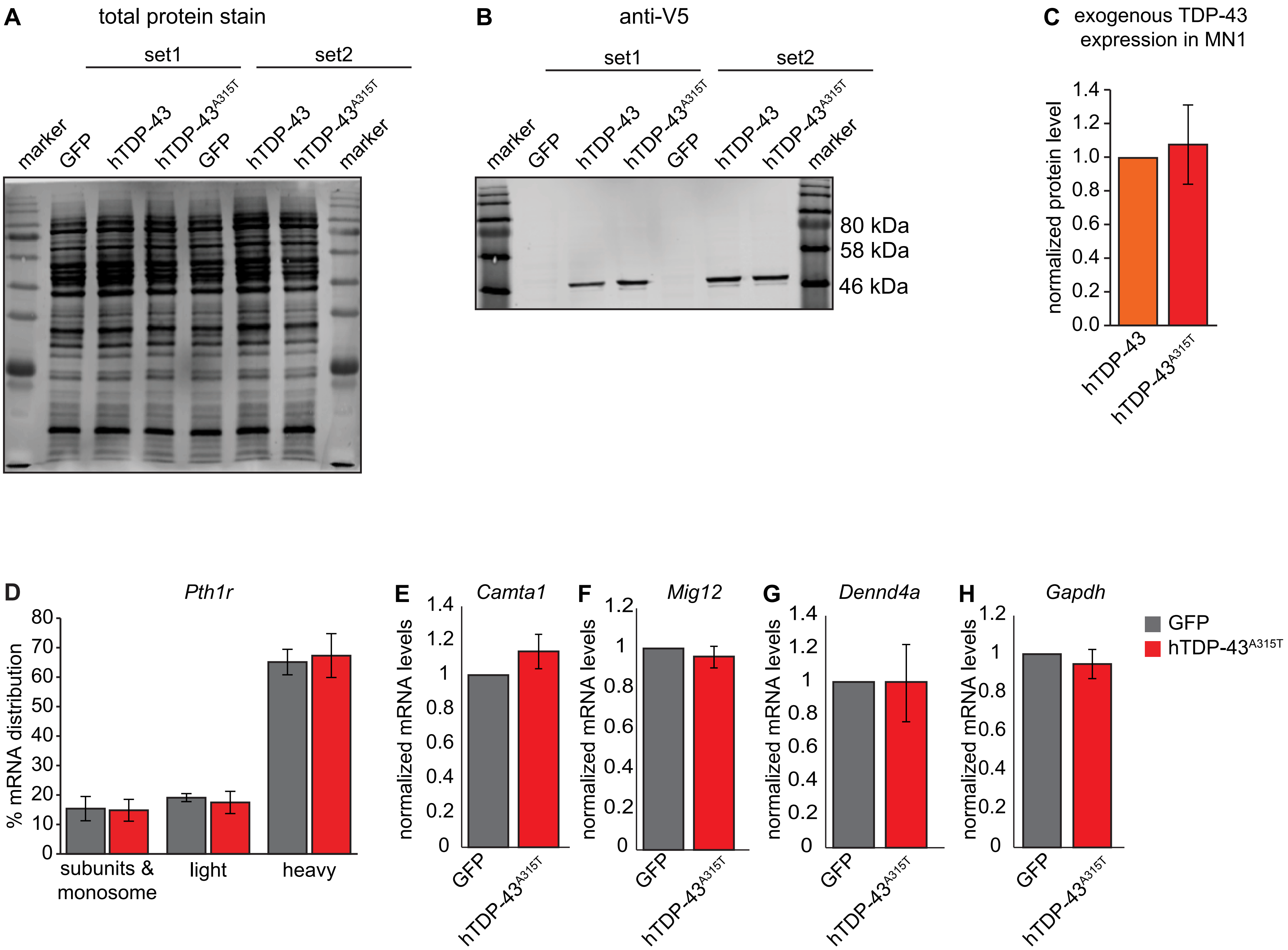

SUPPLEMENTARY FIGURE 3 - Neelagandan et al

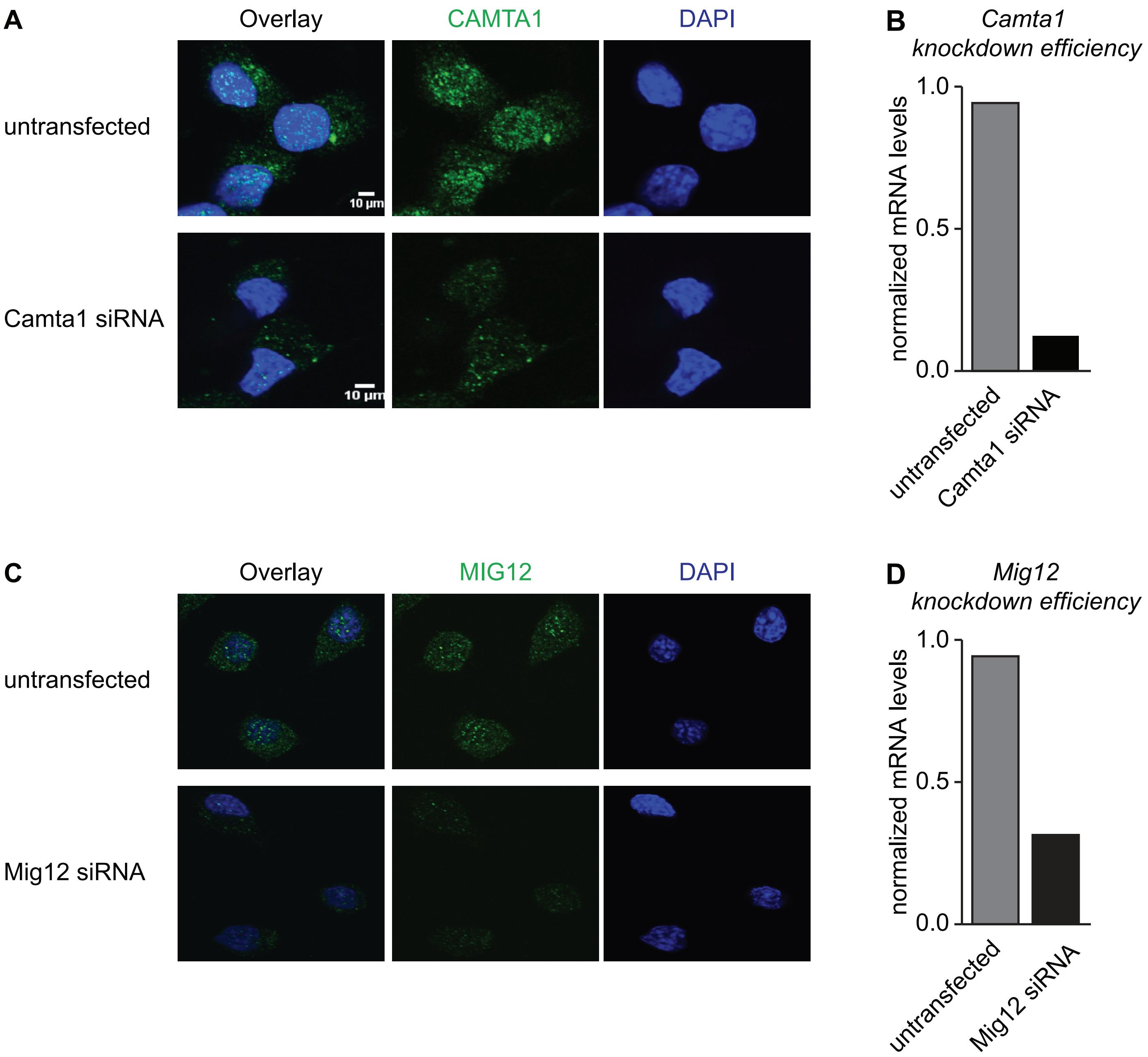

SUPPLEMENTARY FIGURE 4 - Neelagandan et al

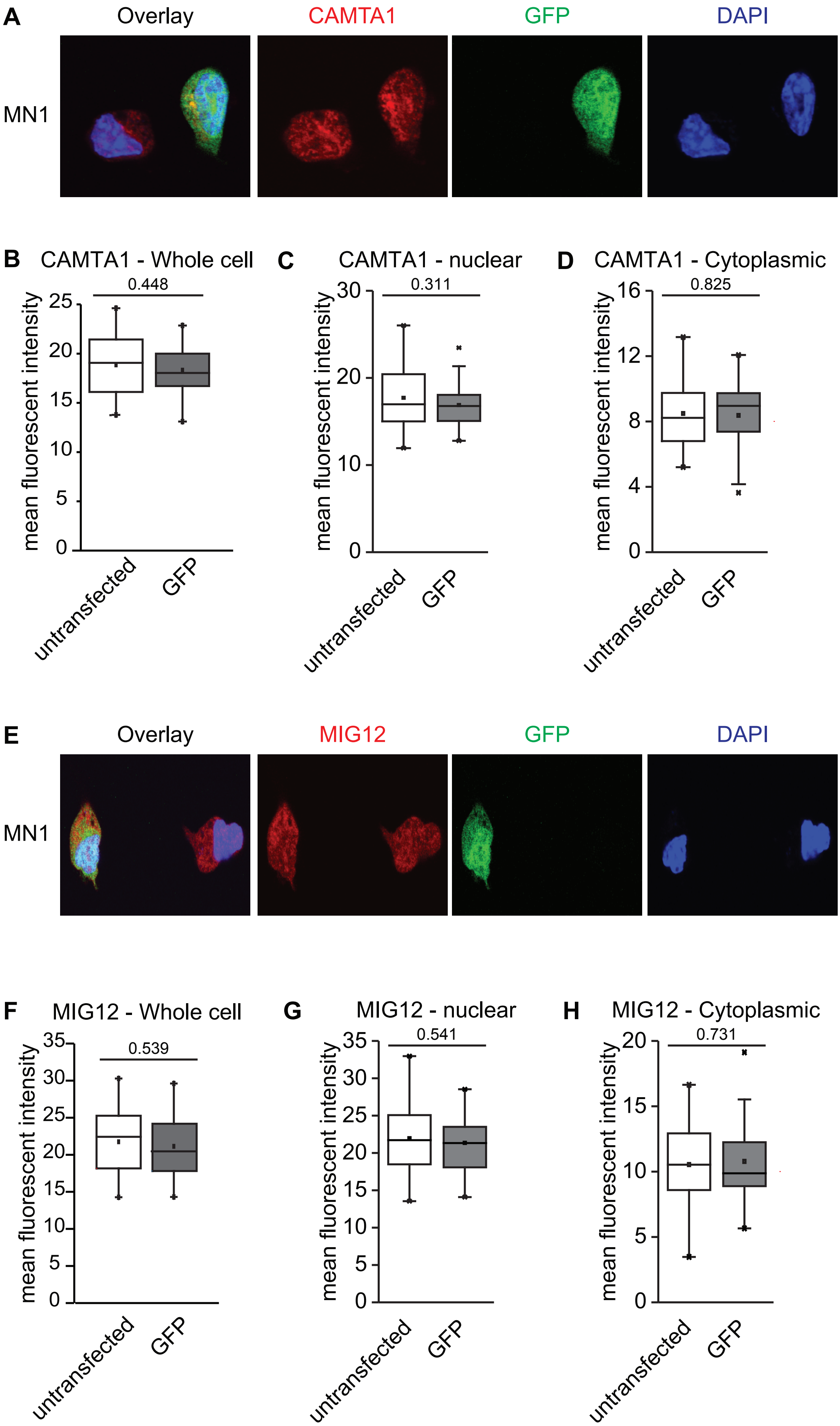

SUPPLEMENTARY FIGURE 5 - Neelagandan et al

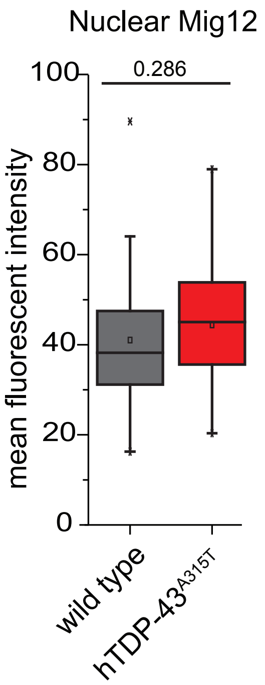

SUPPLEMENTARY FIGURE AND TABLE LEGENDS

Supplementary Figure 1: hTDP-43, hTDP-43^{A315T}, and total TDP-43 protein expression levels in ribosome profiling experiments

(A) Western blot comparing the levels of exogenous protein and endogenous protein in cells transfected with GFP, hTDP-43-V5, or hTDP-43^{A315T}-V5. The blot shows reduction of endogenous TDP-43 upon transfection of hTDP-43 or hTDP-43^{A315T} implying only a modest increase in levels of total TDP-43 in transfected cells. (B) Representative blot showing similar levels of expression of transiently transfected hTDP-43-V5 and hTDP-43^{A315T}-V5 protein in MN1 cells from replicates 1 and 2 used to generate ribosome profiling data. (C) Normalized protein levels of exogenous TDP-43 in transfected MN1 cells, derived from B. (n=2, error bars show deviation from the average for hTDP hTDP-43^{A315T} samples). Note the highly similar expression levels. (D) Representative blot showing expression of transgene-derived hTDP-43 and Flag-hTDP-43^{A315T} protein, as well as total TDP-43 protein in primary cortical neurons used for ribosome footprint profiling. (E) Normalized protein levels of exogenous TDP-43 from all three replicates used to generate ribosome footprint libraries (n=3, s.e.m error bars, *P*-values indicated).

Supplementary Figure 2: hTDP-43-V5 and hTDP-43^{A315T}-V5 expression levels in polysome profiling assays and analysis of mRNA levels for *Camta1*, *Mig12* and *Dennd4a*

(A-B) Immunoblots of transfected MN1 cells for replicates used to generate polysome profiles in Figure 1F-I and Figure 3. (A) Total protein staining. (B) Western blot probed for V5, showing similar expression of exogenous proteins, hTDP-43-V5 and hTDP-43^{A315T}-V5. (C) Normalized protein levels of exogenous TDP-43 obtained from quantification of blot in B normalized to total protein shown in A (n=2, error bars show deviation from average). (D) Plot showing distribution of *Pth1r* mRNA across the gradient. Graphs showing mRNA levels of *Camta1* (E), *Mig12* (F), *Dennd4a* (G) and *Gapdh* (H) in the input material for polysome profiling (n=2, error bars show deviation from average, values normalized to 18s rRNA). Note the similarity of the levels, consistent with the original ribosome profiling data.

Supplementary Figure 3: Validation of CAMTA1 and MIG12 antibodies in immunofluorescence

(A) Immunostaining of MN1 untransfected control and cells transfected with *Camta1* siRNA after 72 hours of knock down stained for CAMTA1 (green) and DAPI (blue). (B) Knockdown efficiency of CAMTA1 measured by qRT-PCR and normalized to *Gapdh*. (C) Immunostaining of MN1 untransfected control and cells transfected with *Mig12* siRNA after 72 hours of knock down stained for MIG12 (green) and DAPI (blue). (D) Knockdown efficiency of MIG12 measured by qRT-PCR and normalized to *Gapdh*. Note the significant decrease in IF signal for both CAMTA1 and MIG12 after siRNA knockdown, implying specific detection of these proteins in MN1 cells under our IF conditions.

Supplementary Figure 4: GFP transfected MN1 cells do not show any changes in CAMTA1 and MIG12 protein levels

(A-D) Immunostaining for CAMTA1 with MN1 cells transfected with pEGFP-C1 plasmid. (A) Representative images showing staining for CAMTA1 (red) and DAPI (blue). Intensity of CAMTA1 in the whole cell (B), nucleus (C), and cytoplasm (D) (n= 30-35 cells each; *P*-value indicated in each plot; unpaired two-tailed t-test). (E-H) Immunostaining for MIG12 with MN1 cells transfected with pEGFP-C1 plasmid. (E) Representative images showing staining for MIG12 (red) and DAPI (blue). Intensity of MIG12 in the whole cell (F), nucleus (G) and in cytoplasm (H) (n= 36, 40 cells; *P*-values indicated in each plot; unpaired two-tailed t-test).

Supplementary Figure 5: MIG12 protein levels do not change in the nucleus of primary cortical neurons expressing TDP-43^{A315T}

Mean fluorescence intensity of MIG12 in the nuclei of DIV-2 primary cortical neurons (two-tailed t-test, *P*-values indicated in the plot).

Supplementary Table 1: Read counts from RNA-Seq and ribosome footprint data sets from MN1 and primary neurons

Supplementary Table 2: Data quality based on read count distribution along 5'UTR, CDS and 3'UTR in total mRNA and footprints for all samples

Supplementary Table 3: List of differentially expressed genes in ribosome footprints from MN1 cells

Supplementary Table 4: List of differentially expressed genes in total mRNA from MN1 cells

Supplementary Table 5: List of differentially expressed genes in ribosome footprints from primary neurons

Supplementary Table 6: List of differentially expressed genes in total mRNA from primary neurons

Supplementary Table 7: CLIP data summary showing C_t values and data analysis for target mRNAs from each biological replicate of CLIP experiments shown in Figure 4

Supplementary Table 8: TDP-43 (UG) repeat motifs in 5'UTR, CDS and 3'UTR of target mRNAs

Supplementary Table 9: Oligos used for cloning
